# Developmental programming in human umbilical cord vein endothelial cells following fetal growth restriction

**DOI:** 10.1186/s13148-020-00980-9

**Published:** 2020-11-30

**Authors:** Fieke Terstappen, Jorg J. A. Calis, Nina D. Paauw, Jaap A. Joles, Bas B. van Rijn, Michal Mokry, Torsten Plösch, A. Titia Lely

**Affiliations:** 1 Division Woman and Baby, Department of Obstetrics, Wilhelmina Children’s Hospital, University Medical Center Utrecht, Postbus 85090, 3508 AB Utrecht, The Netherlands; 2 Department for Developmental Origins of Disease, Wilhelmina Children’s Hospital, University Medical Center Utrecht, Utrecht, The Netherlands; 3 Department of Cardiology, University Medical Center Utrecht, Utrecht, The Netherlands; 4 Center for Translational Immunology, University Medical Centre Utrecht, Utrecht, The Netherlands; 5 Department of Nephrology and Hypertension, University Medical Center Utrecht, Utrecht, The Netherlands; 6 Department of Obstetrics and Fetal Medicine, Erasmus MC University Medical Center Rotterdam, Rotterdam, The Netherlands; 7 Department of Obstetrics and Gynaecology, University Medical Center Groningen, University of Groningen, Groningen, The Netherlands

**Keywords:** Developmental programming, DNA methylation, Epigenetics, Fetal growth restriction, *FPR3*, Gene set enrichment analysis, Human umbilical cord vein endothelial cells, *LGALS1*, *NRM*, RNA-sequencing, Sex differences

## Abstract

**Background:**

Fetal growth restriction (FGR) is associated with an increased susceptibility for various noncommunicable diseases in adulthood, including cardiovascular and renal disease. During FGR, reduced uteroplacental blood flow, oxygen and nutrient supply to the fetus are hypothesized to detrimentally influence cardiovascular and renal programming. This study examined whether developmental programming profiles, especially related to the cardiovascular and renal system, differ in human umbilical vein endothelial cells (HUVECs) collected from pregnancies complicated by placental insufficiency-induced FGR compared to normal growth pregnancies. Our approach, involving transcriptomic profiling by RNA-sequencing and gene set enrichment analysis focused on cardiovascular and renal gene sets and targeted DNA methylation assays, contributes to the identification of targets underlying long-term cardiovascular and renal diseases.

**Results:**

Gene set enrichment analysis showed several downregulated gene sets, most of them involved in immune or inflammatory pathways or cell cycle pathways. seven of the 22 significantly upregulated gene sets related to kidney development and four gene sets involved with cardiovascular health and function were downregulated in FGR (*n* = 11) versus control (*n* = 8). Transcriptomic profiling by RNA-sequencing revealed downregulated expression of *LGALS1*, *FPR3* and *NRM* and upregulation of lincRNA *RP5-855F14.1* in FGR compared to controls. DNA methylation was similar for *LGALS1* between study groups, but relative hypomethylation of *FPR3* and hypermethylation of *NRM* were present in FGR, especially in male offspring. Absolute differences in methylation were, however, small.

**Conclusion:**

This study showed upregulation of gene sets related to renal development in HUVECs collected from pregnancies complicated by FGR compared to control donors. The differentially expressed gene sets related to cardiovascular function and health might be in line with the downregulated expression of *NRM* and upregulated expression of lincRNA *RP5-855F14.1* in FGR samples; NRM is involved in cardiac remodeling, and lincRNAs are correlated with cardiovascular diseases. Future studies should elucidate whether the downregulated *LGALS1* and *FPR3* expressions in FGR are angiogenesis-modulating regulators leading to placental insufficiency-induced FGR or whether the expression of these genes can be used as a biomarker for increased cardiovascular risk. Altered DNA methylation might partly underlie *FPR3* and *NRM* differential gene expression differences in a sex-dependent manner.

## Introduction

Fetal growth restriction (FGR) describes the condition in which the fetus fails to reach its genetically determined growth potential. FGR most commonly results from placental insufficiency, in which a reduced uteroplacental blood flow, oxygen, and nutrients toward the fetus lead to aberrant fetal growth. Compensatory physiological mechanisms are set in motion, such as fetal hemodynamic redistribution over organs and epigenetic alterations; while these fetal adaptations might improve fetal survival, they are considered to be unfavorable in the long run.

FGR is linked to an increased susceptibility for various noncommunicable diseases in adulthood, including cardiovascular and renal disease [1–4]. The Developmental Origins of Health and Disease (DOHaD) hypothesis states that this predisposition originates in the womb, when the adverse in utero environment influences epigenetic developmental programming [5–7]. Preclinical research strongly supports sex-specific programming of cardiovascular and renal disease in FGR offspring [8]. However, evidence for this concept has been less evident in humans [9, 10]. Epigenetic differences have been observed in placental tissue and cord blood collected from pregnancies in which babies were born with a low birth weight (as a surrogate marker for FGR). Most studies investigated DNA methylation and identified epigenetic DNA methylation markers related to impaired fetal growth [11, 12].

While placental tissue can be used to examine gene expression or epigenetic changes in pregnancies complicated by FGR, this tissue consists of a combination of maternal and fetal cells. Therefore, human umbilical vein endothelial cells (HUVECs) can be used to examine the fetal profile of disrupted growth without contamination by maternal cells. Therefore, HUVECs are especially relevant cell type in context of the fetal origin of cardiorenal disease. The few studies performed in cultured HUVECs report different proteome profiles in cultured HUVECs from FGR compared to control donors and differential protein expression and DNA methylation in the eNOS pathway [13–15]. However, transcriptomic profiling by RNA-sequencing of primary HUVECs derived from FGR compared to control donors, without culturing bias, has to our knowledge not yet been reported. In addition, the expression of gene sets related to cardiovascular or renal development and function has not been analyzed in this condition.

This study aims to examine whether developmental programming profiles, especially related to the cardiovascular and renal systems, differ in HUVECs collected from pregnancies complicated by placental insufficiency-induced FGR compared to normal growth pregnancies. We explored this by whole-genome RNA-sequencing to map differential expression per gene and gene set enrichment analysis (GSEA) focussed on cardiovascular and renal development, function and health. Additionally, we performed targeted DNA methylation assays in candidate genes to gain insight in whether DNA methylation plays a regulatory role in the different expression. This approach contributes to the identification of early targets that can be aimed at to predict or prevent long-term diseases.

## Methods

### Study population

Pregnant women with placental insufficiency-induced FGR and pregnant woman with normal grown fetuses were included in this prospective observational study in the Wilhelmina Children’s Hospital from July 2016 to December 2017. Inclusion criteria for placental insufficiency-induced FGR cases were described [16], but in short were diagnosed by prenatal ultrasound when (1) estimated fetal weight or abdominal circumference was below 10^th^ percentile for gestational age, in combination with (2) signs of placental insufficiency defined as abnormal blood flow patterns in umbilical artery, fetal middle cerebral artery, cerebral–placental ratio, or deflecting fetal growth rate in at least three consecutive measurements. The control group consisted of pregnancies with normal fetal growth defined as estimated fetal weight or abdominal circumference between p10–90. Percentiles of prenatal biometry were determined using the perinatology biometry calculator (http://www.perinatology.com/calculators/biometry.htm). Exclusion criteria were congenital disorders, multiple pregnancies and stillbirth. The Medical Ethical Committee of the University Medical Center Utrecht approved the study on July 19, 2016, protocol number 16-302. Written informed consent was obtained from parents prior to delivery.

### Clinical data

Clinical data were derived from electronic patient records (HiX, Chipsoft B.V., the Netherlands). Maternal comorbidities and cardiovascular familiarities included BMI, and smoking, preexisting hypertension, cardiovascular or renal diseases (including congenital disorders), preeclampsia, diabetes, autoimmune disorders. Use of maternal medication was registered. Percentiles for weight and head circumference at birth were determined with Intergrowth-21st [17]. Neonatal complications included infant respiratory distress syndrome, intraventricular hemorrhage, sepsis and necrotizing enterocolitis.

### HUVECs isolation

Directly after placental delivery, the umbilical cord was stored in phosphate buffered saline (PBS) solution (pH 7.2;) at 4 °C. HUVECs isolation occurred preferably within 12 h, but always within 24 h after placental delivery as described [18]. Umbilical cords from *n* = 8 control and *n* = 12 FGR cases were collected. Cannulation of the umbilical vein at one end allowed access to wash with sterile PBS (pH 7.4; Gibco by Life Technologies, Grand Island, NY). Hereafter, the umbilical cord was clamped at both ends in order to incubate with accutase (0.02 µg/ml DNase; Innovative cell technologies Inc, San Diego, CA) for 5 min in sterile PBS at 37 °C to detach the endothelial cells from the umbilical vein. Detached HUVECs in accutase were flushed out of the umbilical vein with endothelial cell growth medium-2 (97% EGM-2; basal medium and SingleQuots supplement [1.9% FBS, 0.04% hydrocortisone, 0.4% hFGF-B2, 0.1% VEGF, 0.1% R3-IGF-1, 0.1% ascorbic acid, 0.1% hEGF, 0.1% GA-1000, 0.1% heparin], Lonza Bioscience, Walkersville, MD) and centrifuged in two separate tubes for 5 min 330 g at room temperature. One pellet was resuspended in 600 μl RA1 lysis buffer (Macherey–Nagel, Düren, Germany) and 6 μl 1 M DTT and stored at − 80 °C until RNA isolation. The second pellet was resuspended in 0.5 ml EMG-2 medium and 0.5 ml freezing medium with 20% DMSO and was frozen in a freezing container overnight and stored in liquid nitrogen the next day until DNA isolation.

### RNA isolation and RNA-sequencing

RNA was isolated using NucleoSpin RNA® (Macherey–Nagel), with RNA elution in 40 μl nuclease-free water. The concentration of RNA was quantified using Qubit RNA HS assay and Qubit fluorometer (Thermo Fisher). RNA-sequencing was performed as described [19]. In short, libraries were generated using NEXTFlexTM Rapid RNA-seq Kit (Bio Scientific) and sequenced by the Nextseq500 platform (Illumina) to produce 75 bp single-end reads through the Utrecht DNA sequencing facility. Reads were aligned to the human reference genome GRCh37 using STAR.

### Gene set analysis

Gene set enrichment testing was performed on the hallmark (H), canonical pathway (C2-CP) and select GO term (C5) gene set collections from the Molecular Signatures Database (version 7.1) [20, 21]. Only gene sets with relation to renal or cardiovascular development, function and health were selected from the GO term gene sets (Additional file 1: Table S1). Gene sets with less than five genes in the set of selected genes (based on expression, see below) were excluded from the analysis, eventually resulting in 2167 included gene sets.

### DNA isolation and methylation

Genomic DNA from HUVEC was isolated with the allprep DNA/RNA mini kit (Qiagen, Venlo, the Netherlands), following the manufacturer’s protocol. DNA quantity was measured with a Nanodrop 2000c (Thermo Scientific, Pittsburgh, PA). DNA was stored at − 80 °C until further analysis.

Targeted DNA methylation assaying was performed blindly in the significant differential expressed genes. Bisulfite conversion of 200 ng DNA was performed with the EZ DNA Methylation-Gold kit (Zymo Research, Leiden, the Netherlands) according to the manufacturers’ protocol. Pyrosequencing primers were designed for the top three differentially expressed genes targeting the promoter regions (Additional file 2: Table S2) using the PyroMark Assay design 2.0 software (Qiagen). HotStarTaq master mix (Qiagen) was used for amplification of 20 ng of bisulfite-treated DNA using the following steps: DNA polymerase activation (95 °C, 15 min), three-step cycle of denaturation (94 °C, 30 s), annealing (*FPR3* 54 °C, *LGALS1* 56 °C, and *NRM* 56 °C; 30 s), and extensions (72 °C, 30 s) repeated for 45 cycles in a row. The final extension was carried out at 72 °C for 7 min.

The polymerase chain reaction product was analyzed for the extent of methylation per selected CpG positions on a PyroMark Q24 (Qiagen). Data were analyzed using the PyroMark Q24 Analysis Software 2.0 (Qiagen).

### Statistical analysis

#### Clinical data

Statistical analysis was performed in IBM SPSS Statistics 25 for Windows, version 25 (IBM Corp, Armonk, NY). Parametric data are presented as mean ± SD and tested with independent t test, nonparametric data are presented as median (minimum–maximum) and tested with Mann–Whitney, and nominal data are presented as *n* (%) and tested with Fisher exact. A two-sided *p* value of ≤ 0.05 was considered significant.

#### Differential expression of genes

Read counts per gene, per sample, were analyzed for global expression differences using R (version 3.5.3). Genes were selected with an expression of one count per million reads (CPM) in at least eight samples (*n* = 13,760 genes selected). Read counts were TMM-normalized using the calcNormFactors function from the edgeR package (version 3.24.3) [22]. TMM-normalized counts were used to assess global transcriptional profile differences of all samples by principal component analysis (PCA). Ten principal components (PC) were analyzed in the PCA analysis; values from each PC were checked for correlation to sample characteristics by the Mann–Whitney *U* test implemented in the SciPy package (version 0.19.0) in python (version 2.7.10). Outliers in RNA-seq data were identified and removed when (1) the number of reads was less than 1.000.000; (2) the number of nonzero genes was less than 10.000 or (3) a combination of number of nonzero genes was between 10.000 and 12.000 and a visible outlier on one of the PCA components. One outlier was identified; thus, *n* = 11 FGR and *n* = 8 control samples were selected for differential expression analysis.

Differential gene expression analysis was performed with the edgeR package (version 3.24.3) in R (version 3.5.3). Gene expression was modeled using the glmQLFit function in EdgeR [22], to a model that included patient group variables, as well as factors to capture Mode of Delivery (caesarean section vs. spontaneous delivery), Sex (male vs. female), and Gestational stage (preterm vs. term)-related gene expression variation. Differential gene expression was determined between study population groups (FGR vs. control). Differential expression statistics were obtained using the glmQLFTest functionality in edgeR; false discovery rates (FDRs) were determined using the Benjamini–Hochberg method to adjust for multiple testing and considered significant when below 0.1 (in combination with *p* value below 0.05) [23].

#### Gene set analysis

Gene set enrichment testing was performed with CAMERA, using the same linear model and contrasts as in the differential gene expression analysis (see above), and FDRs were also determined using the Benjamini–Hochberg method [23]. When a module showed ≥ 50% overlap with a higher ranking gene set, we selected the more significant gene set. Heatmaps for the gene sets related to the cardiovascular and renal development or function were created.

#### DNA methylation

The level of DNA methylation is given as a percentage, and since sex-specific differences have been reported in HUVECs and DNA methylation assays in other reproductive tissue, the data were analyzed with two-way ANOVA with Bonferroni multiple comparison using GraphPad Prism (version 8.4.3, San Diego, California, USA)[18, 24, 25].

## Results

### Study characteristics

Study characteristics are presented in Table 1. There were no maternal cardiovascular diseases diagnosed besides preexisting hypertension or preeclampsia/HELLP. Severe FGR, defined as estimated fetal weight and/or abdominal circumference below the third percentile, was observed in ten out of 11 cases within the FGR group. One out of eight in the control group and four out of 11 in the FGR group were born prematurely. No neonatal death prior to discharged occurred. None of the neonates suffered from necrotizing enterocolitis or sepsis during neonatal intensive care unit admission, interventricular hemorrhage occurred in two control patients, and idiopathic respiratory distress syndrome was diagnosed in one control and one FGR neonate.

**Table 1:** Maternal and neonatal characteristics

	Control (*n* = 8)	FGR (*n* = 11)	*p* value
*Maternal characteristics*			
Age (years)	29 ± 4	32 ± 5	0.11
(Pre-pregnancy) BMI (kg/m^3^)	25 ± 4	25 ± 4	0.96
Preexisting hypertension, *n* (%)	0 (0)	2 (18)	0.49
Renal disease, *n* (%)	0 (0)	1 (9)	1.00
Preexistent diabetes, *n* (%)	1 (13)	0 (0)	0.42
Autoimmune disease, *n* (%)	1 (13)	1 (9)	1.00
Preeclampsia, *n* (%)	0 (0)	5 (46)	*0.05*
HELLP, *n* (%)	0 (0)	1 (9)	1.00
PPROM, *n* (%)	3 (38)	0 (0)	0.06
Smoking, *n* (%)	2 (25)	5 (46)	0.63
*Maternal medication during pregnancy*			
Antihypertensive drugs, *n* (%)	0 (0)	6 (55)	*0.02*
Antenatal steroids, *n* (%)	8 (100)	9 (82)	0.49
MgSO_4,_ *n* (%)	3 (43)^#^	4 (36)	1.00
*Delivery*			
Caesarean section, *n* (%)	2 (25)	7 (64)	0.17
Apgar at 5 min	8 ± 2	8 ± 2	0.28
*Neonatal characteristics*			
Sex, *n* (%male)	3 (38)	6 (55)	0.65
GA at birth (weeks)	31.1 ± 2.6	34.6 ± 3.5	*0.02*
Birth weight (gram)	1681 ± 416	1596 ± 459	0.69
Birth weight (percentile)	66 ± 20	6 ± 12	< *0.01*
- < 3rd percentile, *n* (%)	0 (0)	8 (73)	< *0.01*

Data expressed as mean ± SD or *n* (%), respectively, tested with independent t test or Fisher's exact test. ^#^ represents missing data, and therefore, the percentages are calculated based on the number of observations/measurements within the control group with 7 being the lowest number of patients in a group (maximum 13% missing data). Pre-existing hypertension, preeclampsia and HELLP were defined according to the National Institute for Health and Care Excellence (NICE) guidelines [26]. GA: gestational age; HELLP: hemolysis, elevated liver enzymes and low platelet syndrome; and PPROM: preterm premature rupture of membranes

### Differential expression of genes

Multidimensional scaling (MDS) plots showed clustering in the study population (FGR vs. CON) as potential modifiers, but not in delivery route, prematurity or sex (Additional file 3: Figure S1). PCA plots also showed clustering between FGR vs. CON (Additional file 4: Figure S2). All of the study characteristics were tested for association for all the first ten PCs, and study population was associated with PC1, PC3, PC4 and PC6; delivery route was associated with PC1 and PC7, and gestational stage with PC6 (Additional file 5: Table S3). Therefore, differences in expression due to sex, mode of delivery, and gestational stage were accounted for in the modeling of gene expression.

**Fig. 1:**
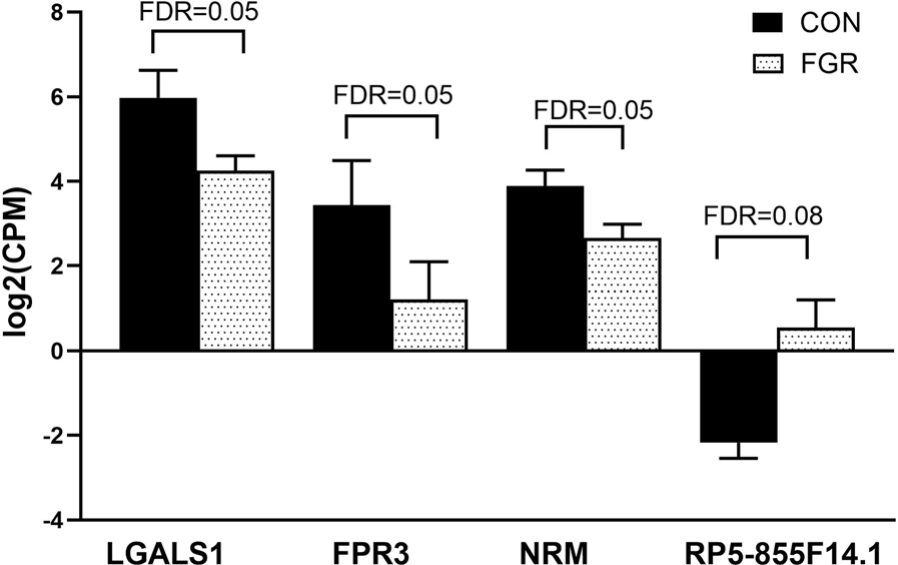
Gene expression values of the genes that significantly differed between fetal growth restriction and control. TMM normalized gene expression of lectin galactoside-binding soluble 1 (*LGALS1*), formyl peptide receptor 3 (*FPR3*), nuclear envelope membrane protein (*NRM*) and *RP5-855F14.1* in human umbilical vein endothelial cells collected from pregnancies complicated by fetal growth restriction (FGR) compared to control (CON). CPM, count per million. Data shown as mean ± SD

Three protein-coding genes and one long intergenic noncoding (linc)RNA gene were significantly regulated (with a FDR < 0.1; Additional file 6: Table S4) in FGR compared with control samples: 1) lectin, galactoside-binding, soluble, 1 (*LGALS1*), 2) formyl peptide receptor 3 (*FPR3*), 3) nurim nuclear envelope membrane protein (*NRM*), 4) lincRNA *RP5-855F14.1*; all protein-coding genes were downregulated and lincRNA gene was upregulated (Fig. 1).

### Differential expression of gene sets

In total, 336 gene sets were significantly different between FGR and CON (with a FDR < 0.1; Additional file 7: Table S5). Selection of only the highest ranking gene set module (overlapping modules excluded) resulted in 193 downregulated and 22 upregulated gene sets in FGR versus control samples. The downregulated gene sets are mostly involved with immune, inflammatory or cell cycle pathways. As we were interested in risk of developing cardiovascular or renal disease, we noticed that the several gene sets related to renal development were significantly upregulated and a few related to cardiovascular health and function were downregulated in FGR samples vs. CON samples (Table 2). Heatmaps were made to study the extent of up and downregulation for the distinct genes in these gene sets in each sample (Additional file 8: Figure S3). From this analysis, most genes were up- and downregulated in accordance with the differential gene set analysis results.

**Table 2:** Significantly different gene sets related to renal and cardiovascular development, function or health

Gene set name	Up or down	*p* value	FDR	Brief description
KEGG_CARDIAC_MUSCLE_CONTRACTION	Down	0.0002	0.0052	Contraction of the heart is a complex process initiated by the electrical excitation of cardiac myocytes
GO_KIDNEY_EPITHELIUM_DEVELOPMENT	Up	0.0003	0.0080	The process whose specific outcome is the progression of an epithelium in the kidney over time, from its formation to the mature structure
GO_RENAL_FILTRATION	Up	0.0003	0.0081	Renal system process in which fluid circulating through the body is filtered through a barrier system
GO_RENAL_SYSTEM_VASCULATURE_DEVELOPMENT	Up	0.0007	0.0126	The process whose specific outcome is the progression of vasculature of the renal system over time, from its formation to the mature structure
GO_CARDIAC_SEPTUM_DEVELOPMENT	Up	0.0009	0.0145	The progression of a cardiac septum over time, from its initial formation to the mature structure
REACTOME_ERYTHROPOIETIN_ACTIVATES_PHOSPHOINOSITIDE_3_KINASE_PI3K	Down	0.0012	0.0172	Erythropoietin activates phosphoinositide-3-kinase
GO_RENAL_SYSTEM_DEVELOPMENT	Up	0.0015	0.0207	The process whose specific outcome is the progression of the renal system over time, from its formation to the mature structure
REACTOME_SIGNALING_BY_ERYTHROPOIETIN	Down	0.0026	0.0296	Signaling by erythropoietin
GO_KIDNEY_MESENCHYME_DEVELOPMENT	Up	0.0042	0.0426	The biological process whose specific outcome is the progression of a kidney mesenchyme from an initial condition to its mature state. This process begins with the formation of kidney mesenchyme and ends with the mature structure
REACTOME_CELL_SURFACE_INTERACTIONS_AT_THE_VASCULAR_WALL	Down	0.0067	0.060	Cell surface interactions at the vascular wall
REACTOME_SYNTHESIS_OF_VERY_LONG_CHAIN_FATTY_ACYL_COAS	Down	0.0079	0.0671	Synthesis of very long-chain fatty acyl-CoAs
GO_CELL_DIFFERENTIATION_INVOLVED_IN_KIDNEY_DEVELOPMENT	Up	0.0088	0.0712	The process in which relatively unspecialized cells acquire specialized structural and/or functional features that characterize the cells of the kidney as it progresses from its formation to the mature state
GO_REGULATION_OF_GLOMERULAR_FILTRATION	Up	0.0115	0.0847	Any process that modulates the frequency, rate or extent of glomerular filtration. Glomerular filtration is the process in which blood is filtered by the glomerulus into the renal tubule
REACTOME_TRIGLYCERIDE_METABOLISM	Down	0.0141	0.0947	Triglyceride metabolism

Ordered according to lowest false discovery rate (FDR)

### DNA methylation

**Fig. 2:**
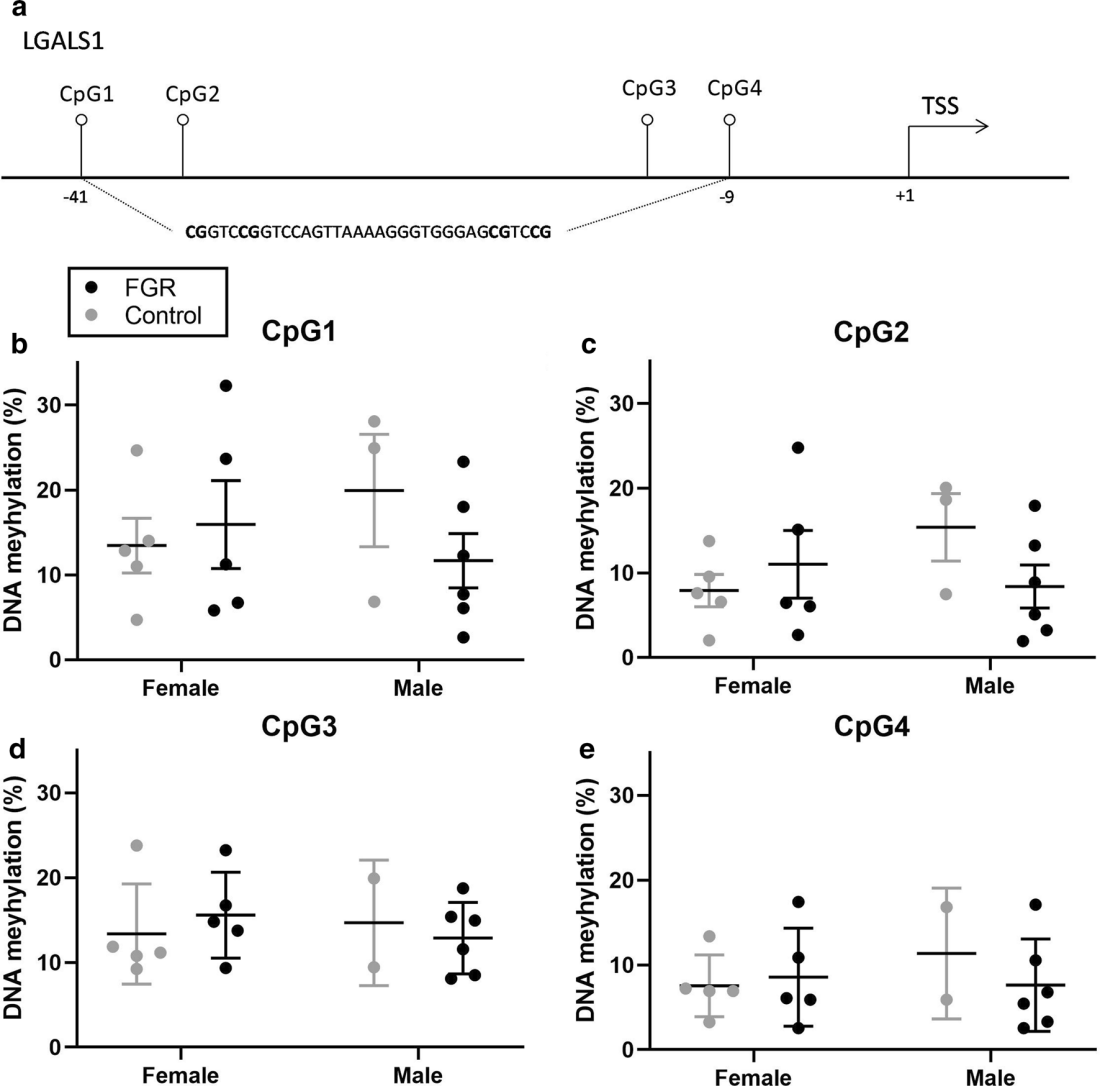
DNA methylation at individual CpG positions for *LGALS1*. **a** The examined CpG positions in relation to the transcription start site (TSS); **b** DNA methylation at CpG1; **c** DNA methylation at CpG2; **d** DNA methylation at CpG3; **e** DNA methylation at CpG4 in fetal growth restriction (FGR) (*n* = 11) vs. control (*n* = 8). Data shown as Mean ± SD. Tested with two-way ANOVA with Bonferroni multiple comparison. *LGALS1,* lectin galactoside-binding soluble 1

**Fig. 3:**
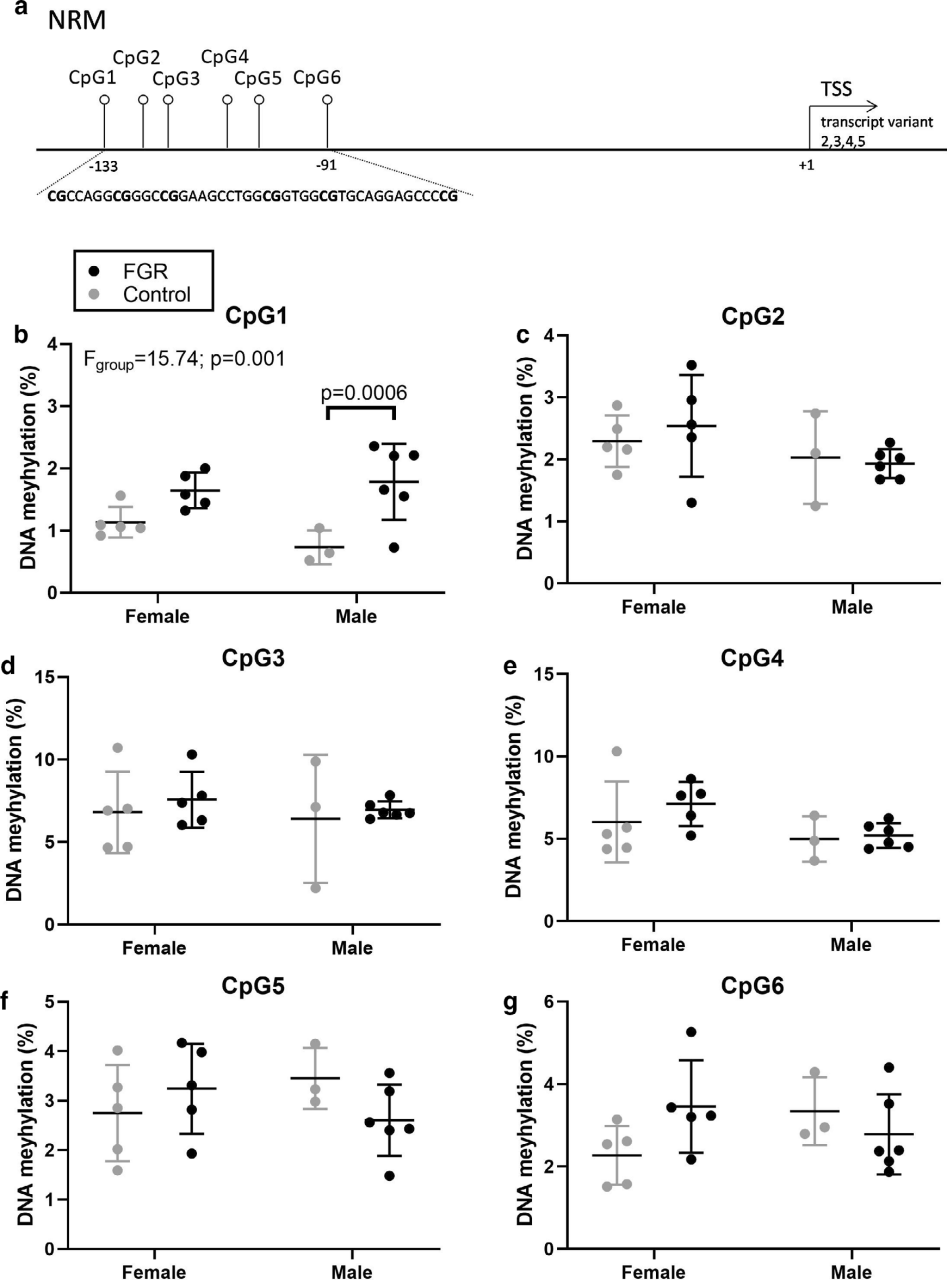
DNA methylation at individual CpG positions for *NRM*. **a** The examined CpG positions in relation to the transcription start site (TSS); **b** DNA methylation at CpG1; **c** DNA methylation at CpG2; **d** DNA methylation at CpG3; **e** DNA methylation at CpG4; **f** DNA methylation at CpG5; **g** DNA methylation at CpG6 in fetal growth restriction (FGR) (*n* = 11) vs. control (*n* = 8). Data shown as mean ± SD. Tested with two-way ANOVA with Bonferroni multiple comparison. *NRM,* nurim nuclear envelope membrane protein

The percentage methylation was measured at each individual CpG position for selected areas of the promoters of the three protein-encoding genes *LGALS1*, *FPR3* and *NRM* per study population (Table 3). *LGALS1* showed similar percentage of methylation at each individual CpG position between groups, independent of sex (Fig. 2). DNA hypomethylation differed between in FGR males versus CON males at CpG2 of *FPR3* only (Additional file 9: Figure S4). *NRM* was significantly hypermethylated at CpG1 in FGR compared to control, especially in male FGR offspring (Fig. 3).

**Table 3:** DNA methylation of each CpG position for the three highest significantly different expressed genes

Gene	CpG position	Methylation (%) Control (*n* = 8)	Methylation (%) FGR (*n* = 11)	*p* value
*LGALS1*	CpG1	15.88 ± 8.87	13.61 ± 9.44	0.60
	CpG2	10.71 ± 6.24	9.58 ± 7.30	0.73
	CpG3	11.16 (9.22–23.78)^†^	14.80 (8.08–23.24)	0.86
	CpG4	8.63 ± 4.71^†^	8.05 ± 5.32	0.82
*FPR3*	CpG1	93.55 ± 1.21	92.60 ± 1.24	0.11
	CpG2	96.15 ± 1.31	94.68 ± 2.14	0.10
*NRM*	CpG1	0.98 ± 0.31	1.72 ± 0.47	*0.001*
	CpG2	2.20 ± 0.53	2.21 ± 0.63	0.96
	CpG3	6.97 (2.21–10.71)	6.78 (6.03–10.30)	0.72
	CpG4	5.09 (3.67–10.31)	5.76 (4.39–8.63)	0.31
	CpG5	3.01 ± 0.88	2.89 ± 0.84	0.77
	CpG6	2.68 ± 0.89	3.09 ± 1.05	0.38

Data expressed as mean ± SD tested with independent t test or median (min–max) tested with Mann–Whitney. ^†^7 instead of eight samples

## Discussion

This study examined developmental cardiovascular and renal programming profiles in HUVECs collected from pregnancies complicated by placental insufficiency-induced FGR compared to normal growth pregnancies to identify targets underlying long-term cardiovascular and renal diseases. We report downregulated expression of the protein-coding genes *LGALS1*, *FPR3* and *NRM* and upregulation of the lincRNA *RP5-855F14.1* in FGR samples compared to controls. Sex-dependent DNA methylation might partially underlie *FPR3* and *NRM* gene expression, but we did not observe this for *LGALS1*. Additionally, of the significantly differentially expressed gene sets, the downregulated ones were mostly involved with immune, inflammatory, or cell cycle pathways. Interestingly, seven of the 22 significantly upregulated gene sets related to kidney development and four gene sets related to cardiovascular function and health differed between FGR and control.

### Downregulated expression of *LGALS1*

*LGALS1* is the protein coding gene for galectin-1 (Gal-1) [27]. During pregnancy, Gal-1 is important for immunomodulatory and vascular adaptions required for healthy placentation [28–30]. In HUVECs, Gal-1 mediates angiogenesis via vascular endothelial growth factor receptor (VEGFR)-2 but also the neuropilin receptor (NPR)-1 which enhances the binding between VEGF and VEGFR-2 [29, 31]. Freitag et al*.* also showed that *LGALS1* expression was downregulated in placentas derived from early-onset preeclamptic patients, which is a placental insufficiency syndrome just as in FGR and part of our study population [30]. In addition, in a mouse model inhibition of Gal-1 mediated angiogenesis resulted in preeclamptic symptoms and fetal growth restriction [30]. However, higher or no difference in Gal-1 expression was observed in term placentas from pregnancies complicated by respectively preeclampsia or FGR [32, 33]. Considering the different histopathology between early-onset and late-onset FGR (or preeclampsia), the downregulated *LGALS1* expression in early-onset FGR speculatively contributes directly to placental insufficiency, while the upregulated expression observed in late-onset FGR might be secondary to relative placental–umbilical hypoxia or reduced umbilical flow [30, 32, 34]. Gal-1 has been suggested as an early marker of endothelial dysfunction, and dysregulated Gal-1 has been linked to poor blood pressure regulation and development of cardiovascular disease [27, 28]. Therefore, the finding of downregulated *LGALS1* expression in our FGR samples might be the key regulator leading to placental insufficiency-induced FGR, as well as an indication for the possible higher risk of developing long-term cardiovascular dysfunction in these offspring. Epigenetic processes or post-transcriptional modifications other than DNA methylation might be involved in reduced *LGALS1* expression.

### Downregulated expression of *FPR3* and *NRM *and upregulation of *lincRNA RP5-855F14.1*

In contrast to *LGALS1*, *FPR3*, *NRM,* and *RP5-855F14.1* are less studied genes, especially in the context of FGR and pregnancy. To our knowledge, this is the first study reporting downregulated expression of *FPR3* and *NRM* and upregulation of *RP5-8551F14.1* in HUVECs or other pregnancy tissues collected from pregnancies complicated by FGR. One study in mice suggested that NRM might be involved in early cardiac development [35]. While lincRNAs in general have been described to be epigenetic process-associated with cardiovascular disease and development, the exact function of this specific lincRNA is unknown [24, 36]. In humans, circulating FPR3 mRNA in combination with three other circulating mRNA had a high specificity and sensitivity to predict early-onset PE [37], but little is known on *FPR3* expression or function. Recent studies did link downregulated expression of *FPR2*—another member of the FPR family with high analogies in sequencing identity and downstream responses [38]—in placental tissue to endothelial dysfunction and placental insufficiency via impaired immunomodulatory and angiogenic processes, leading to FGR [39, 40]. FPR2 has also been described to play a protective and repairing role in ischemic heart disease and stroke [41, 42]. Considering the similarities between FPR2 and FPR3 [38], the observed downregulation of *FPR3* in our placental insufficiency-induced HUVECs samples could potentially contribute to placental insufficiency in a similar manner and dysregulated FPR3 might contribute to the increased susceptibility to cardiovascular disease.

DNA methylation might partly contribute to the differential gene expression patterns of *FPR3* and *NRM* in a sex-dependent manner. The sex dependency is especially interesting given that hypertension is more pronounced in fetal growth restricted male offspring [6, 8, 43]. However, while the methylation differences are significant for *NRM*, they are relatively small suggesting that the regulating role of DNA methylation in *NRM* expression is not strong. In addition, *FPR3* is expressed, while the CpGs show hypermethylation; since the closest CpG position is almost 400 bp upstream of the TSS (although not by exclusion), its methylation possibly has little regulatory effects.

### Cardiovascular and renal gene sets

We focused our gene set analysis on cardiovascular and renal development and function, since FGR has been associated with increased susceptibility to develop cardiovascular and renal disease. The four different cardiovascular gene sets, including lipid metabolism, might be in line with the cardiovascular risk profile described with dysregulated expression of *LGALS1* and *RP5-8551F14.1*. While the gene sets related to cardiovascular development were similar in both groups, several gene sets related to kidney development were relatively upregulated in FGR. Nephrogenesis starts around 22 days of gestation and is complete at 34–36 weeks of gestation, making pregnancy the most vulnerable period to impact nephron endowment [44]. FGR has been linked to reduced nephron count and morphological differences in glomeruli, which could lead to glomerular hypertension and compensatory hyperfiltration in the remaining nephrons, causing subsequent nephron loss (Brenner hypothesis) [45]. A recent study using three different rat models for FGR also reported that molecular pathways differed in kidneys from FGR and control male offspring at birth and at postnatal day seven (end of nephrogenesis in rats) [46]. The pathways involved depended on the stage of development, and most upregulation was observed in the placental insufficiency-induced model (best matching our study population). The upregulation of the gene sets related to kidney development in our study, although not evident at individual gene level, suggests that developmental programming difference as a consequence of FGR is a subtle process. In an animal study, upregulation of renal genes in combination with wider nephrogenic zones suggested delayed nephrogenesis in FGR [47]; however, due to ethical reasons, we cannot confirm this histologically. Therefore, whether the upregulation in HUVECs represents accelerated or delayed kidney development and whether this relates to long-term renal function or disease remain to be elucidated.

### Strengths and limitations

This is the first study using HUVECs to investigate developmental programming differences between placental insufficiency-induced FGR and controls by a full-transcriptome RNA-sequencing approach followed by differential gene and gene set expression analysis and follow-up DNA methylation assaying. The gene set analysis focused on cardiovascular and renal development, to specifically test the hypothesis that this correlate with increased risk for diseases of these systems can already be found during pregnancy. The transcriptomic profiling by RNA-sequencing enabled us to find FGR associated gene expression differences in an unsupervised manner, and to select genes for the analysis of DNA methylation. A major strength is that we used prenatal ultrasound measurements to clearly define the placental insufficiency-induced FGR phenotype in our study population; most studies use birth weight as surrogate marker, but this umbrella term also includes other underlying mechanisms such as congenital disorders or constitutionally small children, which have not been exposed to a hostile in utero environment. The strength of investigating HUVECs is that they allow us to investigate fetal expression differences without the interference of maternal cells. Additional strengths are that we did not culture our collected HUVECs and that we isolated the HUVECs within 24 h after delivery which limits external influences on epigenetic results.

While our strictly defined study population created a clear placental insufficiency-induced FGR phenotype, it also limited the number of included samples. However, (post hoc) power calculations for RNA-seq data are not well developed and as such not custom to use for this type of analysis. Instead, we relied on multiple testing corrects, and variation estimates build into the edgeR pipeline to properly analyze our data. Although beyond the scope of our study, we acknowledge that we cannot correlate gene expression findings to long-term outcomes. Gestational age at birth differed between groups, but we accounted for this factor in modeling gene expression and plots of gene expression per sample versus gestational age at birth showed that this was not of influence. The downside of not culturing our HUVECs was that the yielded RNA concentrations were relatively low. However, careful consideration of the number of reads and nonzero genes in all samples allowed detection and exclusion of low-quality samples. Despite extensive washing, HUVEC samples might have been contaminated with a few other fetal blood cells. Other (epi)genetic mechanisms besides DNA methylation possibly involved in differentially regulated expression of genes were not tested. Confirmation of our programming hypothesis includes verification in other tissues.

## Summary and future perspectives

In conclusion, this study showed downregulated expression of *LGALS1*, *FPR3* and *NRM* and upregulation of *RP5-855F14.1* in HUVECs collected from placental insufficiency-induced FGR compared to control. Additionally, several gene sets related to kidney development were upregulated and a few gene sets related to cardiovascular risk were downregulated in FGR. How these findings correlate with long-term cardiovascular and renal function requires further investigation and follow-up studies. The differentially expressed genes (or their encoded protein) might be used as a biomarker, which could contribute to personalized care by predicting the risk of developing cardiorenal disease and selective follow-up of only the patients at risk. Further studies are also required to elucidate how and whether the downregulation of *LGALS1* and *FPR3* are causal regulators resulting in placental insufficiency-induced early-onset FGR as they might hold promise as potential novel targets for preventive treatment. While the findings of this study are the first to support the concept of developmental epigenetic programming of cardiorenal disease following placental insufficiency-induced FGR in humans, they are also merely the first of many steps toward clinical applicability.

## Supplementary information

**Additional file 1**. Table S1: Overview of the gene sets related to renal and cardiovascular development, function and health.

**Additional file 2**. Table S2: primers targeting promotor regions.

**Additional file 3**. Figure S1: multidimensional scaling (MDS) plots.

**Additional file 4**. Figure S2: principal component analysis (PCA) plots of fetal growth restriction (FGR) vs. control (CTRL) .

**Additional file 5**. Table S3: per principal component best correlated modulator.

**Additional file 6**. Table S4: differential expression of FGR versus control (FGR–control).

**Additional file 7**. Table S5: All MsigDB gene modules, as determined by CAMERA, in gene list ranked based on a comparison of: differential expression of FGR versus control (FGR–control). Modules sorted by significance.

**Additional file 8**. Figure S3: heatmaps of significantly differential expressed gene sets involved in cardiovascular or renal development and disease.

**Additional file 9**. Figure S4: DNA methylation at individual CpG positions for FPR3.

## Data Availability

The datasets generated and/or analyzed during the current study are not publicly available due to the Dutch privacy law to protect participants, but are partly and always coded available from the corresponding author on request. All data generated or analyzed during this study are included in this published article and its supplementary information files.

## Abbreviations

CON: Control
CPM: Count per million reads
CTRL: Control
DOHaD: Developmental Origins of Health and Disease
FGR: Fetal growth restriction
*FPR3*: Formyl peptide receptor 3
GSEA: Gene set enrichment analysis
HUVECs: Human umbilical vein endothelial cells
*LGALS1*: Lectin galactoside-binding soluble 1
*NRM*: Nuclear envelope membrane protein
SD: Standard deviation
VEGFR: Vascular endothelial growth factor receptor
