# Supplementary material for: Developmental programming in human umbilical cord vein endothelial cells following fetal growth restriction
Source: Clin Epigenetics. 2020 Nov 30;12:185. doi: 10.1186/s13148-020-00980-9 (PMC7708922; doi:10.1186/s13148-020-00980-9)
Supplement: Supplementary file 1 — Additional file 1. Table S1: Overview of the gene sets related to renal and cardiovascular development, function and health. [file 13148_2020_980_MOESM1_ESM.docx]

**Table S1: Overview of the gene-sets related to renal and cardiovascular development, function and health**

| **Gene-sets involved with cardiovascular development, function, and health** |
| --- |
| KEGG_CARDIAC_MUSCLE_CONTRACTION |
| REACTOME_PLATELET_ACTIVATION_SIGNALING_AND_AGGREGATION |
| BIOCARTA_GCR_PATHWAY |
| BIOCARTA_NO1_PATHWAY |
| KEGG_VASCULAR_SMOOTH_MUSCLE_CONTRACTION |
| BIOCARTA_CARDIACEGF_PATHWAY |
| KEGG_ARRHYTHMOGENIC_RIGHT_VENTRICULAR_CARDIOMYOPATHY_ARVC |
| BIOCARTA_P53HYPOXIA_PATHWAY |
| BIOCARTA_NFAT_PATHWAY |
| BIOCARTA_HIF_PATHWAY |
| KEGG_HYPERTROPHIC_CARDIOMYOPATHY_HCM; KEGG_DILATED_CARDIOMYOPATHY |
| REACTOME_CELL_SURFACE_INTERACTIONS_AT_THE_VASCULAR_WALL |
| KEGG_VEGF_SIGNALING_PATHWAY |
| BIOCARTA_PAR1_PATHWAY |
| BIOCARTA_PLATELETAPP_PATHWAY |
| BIOCARTA_PGC1A_PATHWAY |
| BIOCARTA_ALK_PATHWAY |
| BIOCARTA_AMI_PATHWAY |
| HALLMARK_ANGIOGENESIS |
| REACTOME_ENOS_ACTIVATION_AND_REGULATION |
| REACTOME_HEMOSTASIS |
| REACTOME_VEGF_LIGAND_RECEPTOR_INTERACTIONS |
| REACTOME_REGULATION_OF_HYPOXIA_INDUCIBLE_FACTOR_HIF_BY_OXYGEN |
| BIOCARTA_VEGF_PATHWAY |
| BIOCARTA_HIF_PATHWAY |
| PID_ENDOTHELIN_PATHWAY |
| PID_HIF1_TFPATHWAY |
| PID_HIF1A_PATHWAY |
| PID_HIF2PATHWAY |
| PID_VEGF_VEGFR_PATHWAY |
| PID_VEGFR1_2_PATHWAY |
| PID_VEGFR1_PATHWAY |
| PID_THROMBIN_PAR1_PATHWAY |
| PID_THROMBIN_PAR4_PATHWAY |
| REACTOME_FORMATION_OF_FIBRIN_CLOT_CLOTTING_CASCADE |
| REACTOME_SIGNALING_BY_VEGF |
| REACTOME_METABOLISM_OF_LIPIDS |
| BIOCARTA_LDL_PATHWAY |
| HALLMARK_FATTY_ACID_METABOLISM |
| GO_ATRIAL_CARDIAC_MUSCLE_TISSUE_DEVELOPMELOPMENT |
| GO_ATRIOVENTRICULAR_CANAL_DEVELOPMENT |
| GO_BRANCHING_INVOLVED_IN_BLOOD_VESSEL_MORPHOGENESIS |
| GO_CARDIAC_ATRIUM_DEVELOPMENT |
| GO_CARDIAC_CELL_DEVELOPMENT |
| GO_CARDIAC_CELL_FATE_COMMITMENT |
| GO_CARDIAC_CHAMBER_DEVELOPMENT |
| GO_CARDIAC_CHAMBER_FORMATION |
| GO_CARDIAC_CHAMBER_MORPHOGENESIS |
| GO_CARDIAC_CONDUCTION_SYSTEM_DEVELOPMENT |
| GO_CARDIAC_EPITHELIAL_TO_MESENCHYMAL_TRANSITION |
| GO_CARDIAC_LEFT_VENTRICLE_MORPHOGENESIS |
| GO_CARDIAC_MUSCLE_CELL_CARDIAC_MUSCLE_CELL_ADHESION |
| GO_CARDIAC_MUSCLE_CELL_CONTRACTION |
| GO_CARDIAC_MUSCLE_CELL_DIFFERENTIATION |
| GO_CARDIAC_MUSCLE_CELL_MYOBLAST_DIFFERENTIATION |
| GO_CARDIAC_MUSCLE_CELL_PROLIFERATION |
| GO_CARDIAC_MUSCLE_CONTRACTION |
| GO_CARDIAC_MUSCLE_FIBER_DEVELOPMENT |
| GO_CARDIAC_MUSCLE_MYOBLAST_PROLIFERATION |
| GO_CARDIAC_MUSCLE_TISSUE_DEVELOPMENT |
| GO_CARDIAC_MUSCLE_TISSUE_MORPHOGENESIS |
| GO_CARDIAC_MUSCLE_TISSUE_REGENERATION |
| GO_CARDIAC_MYOFIBRIL |
| GO_CARDIAC_SEPTUM_DEVELOPMENT |
| GO_CARDIAC_NEURAL_CREST_CELL_DEVELOPMENT_INVOLVED_IN_OUTFLOW_TRACT_MORPHOGENESIS |
| GO_CARDIAC_NEURAL_CREST_CELL_DIFFERENTIATION_INVOLVED_IN_HEART_DEVELOPMENT |
| GO_CARDIAC_PACEMAKER_CELL_DIFFERENTIATION |
| GO_CARDIAC_RIGHT_VENTRICLE_MORPHOGENESIS |
| GO_CARDIAC_SEPTUM_MORPHOGENESIS |
| GO_CARDIAC_VASCULAR_SMOOTH_MUSCLE_CELL_DIFFERENTIATION |
| GO_CARDIAC_VENTRICLE_DEVELOPMENT |
| GO_CARDIAC_VENTRICLE_FORMATION |
| GO_CARDIAC_VENTRICLE_MORPHOGENESIS |
| GO_CARDIOBLAST_DIFFERENTIATION |
| GO_CARDIOBLAST_PROLIFERATION |
| GO_CARDIOCYTE_DIFFERENTIATION |
| GO_CARDIOVASCULAR_SYSTEM_DEVELOPMENT |
| GO_CELLULAR_RESPONSE_TO_VASCULAR_ENDOTHELIAL_GROWTH_FACTOR_STIMULUS |
| GO_ENDOCARDIAL_CUSHION_DEVELOPMENT |
| GO_EPITHELIAL_TO_MESENCHYMAL_TRANSITION_INVOLVED_IN_ENDOCARDIAL_CUSHION_FORMATION |
| GO_HEART_FORMATION |
| GO_HIS_PURKINJE_SYSTEM_DEVELOPMENT |
| GO_INTERCALATED_DISC |
| GO_LEUKOCYTE_ADHESION_TO_VASCULAR_ENDOTHELIAL_CELL |
| GO_MUSCLE_HYPERTROPHY |
| GO_NEGATIVE_REGULATION_OF_CARDIAC_MUSCLE_ADAPTATION |
| GO_NEGATIVE_REGULATION_OF_CARDIAC_MUSCLE_CELL_PROLIFERATION |
| GO_NEGATIVE_REGULATION_OF_CARDIAC_MUSCLE_CONTRACTION |
| GO_NEGATIVE_REGULATION_OF_CARDIAC_MUSCLE_TISSUE_DEVELOPMENT |
| GO_NEGATIVE_REGULATION_OF_CARDIAC_MUSCLE_TISSUE_GROWTH |
| GO_NEGATIVE_REGULATION_OF_CARDIOCYTE_DIFFERENTIATION |
| GO_NEGATIVE_REGULATION_OF_CELL_GROWTH_INVOLVED_IN_CARDIAC_MUSCLE_CELL_DEVELOPMENT |
| GO_NEGATIVE_REGULATION_OF_CELLULAR_RESPONSE_TO_VASCULAR_ENDOTHELIAL_GROWTH_FACTOR_STIMULUS |
| GO_NEGATIVE_REGULATION_OF_VASCULAR_ASSOCIATED_SMOOTH_MUSCLE_CELL_APOPTOTIC_PROCESS |
| GO_NEGATIVE_REGULATION_OF_VASCULAR_ASSOCIATED_SMOOTH_MUSCLE_CELL_MIGRATION |
| GO_NEGATIVE_REGULATION_OF_VASCULAR_ENDOTHELIAL_CELL_PROLIFERATION |
| GO_NEGATIVE_REGULATION_OF_VASCULAR_ENDOTHELIAL_GROWTH_FACTOR_RECEPTOR_SIGNALING_PATHWAY |
| GO_NEGATIVE_REGULATION_OF_VASCULAR_SMOOTH_MUSCLE_CELL_DIFFERENTIATION |
| GO_NEGATIVE_REGULATION_OF_VASCULAR_SMOOTH_MUSCLE_CELL_PROLIFERATION |
| GO_PHYSIOLOGICAL_CARDIAC_MUSCLE_HYPERTROPHY |
| GO_POSITIVE_REGULATION_OF_CARDIAC_MUSCLE_ADAPTATION |
| GO_POSITIVE_REGULATION_OF_CARDIAC_MUSCLE_CELL_DIFFERENTIATION |
| GO_POSITIVE_REGULATION_OF_CARDIAC_MUSCLE_CELL_PROLIFERATION |
| GO_POSITIVE_REGULATION_OF_CARDIAC_MUSCLE_TISSUE_DEVELOPMENT |
| GO_POSITIVE_REGULATION_OF_CARDIAC_VASCULAR_SMOOTH_MUSCLE_CELL_DIFFERENTIATION |
| GO_POSITIVE_REGULATION_OF_CARDIOBLAST_DIFFERENTIATION |
| GO_POSITIVE_REGULATION_OF_CARDIOCYTE_DIFFERENTIATION |
| GO_POSITIVE_REGULATION_OF_CELL_GROWTH_INVOLVED_IN_CARDIAC_MUSCLE_CELL_DEVELOPMENT |
| GO_POSITIVE_REGULATION_OF_CELL_MIGRATION_BY_VASCULAR_ENDOTHELIAL_GROWTH_FACTOR_SIGNALING_PATHWAY |
| GO_POSITIVE_REGULATION_OF_ENDOTHELIAL_CELL_CHEMOTAXIS_BY_VEGF_ACTIVATED_VASCULAR_ENDOTHELIAL_GROWTH_FACTOR_RECEPTOR_SIGNALING_PATHWAY |
| GO_POSITIVE_REGULATION_OF_VASCULAR_ASSOCIATED_SMOOTH_MUSCLE_CELL_APOPTOTIC_PROCESS |
| GO_POSITIVE_REGULATION_OF_VASCULAR_ASSOCIATED_SMOOTH_MUSCLE_CELL_MIGRATION |
| GO_POSITIVE_REGULATION_OF_VASCULAR_ENDOTHELIAL_CELL_PROLIFERATION |
| GO_POSITIVE_REGULATION_OF_VASCULAR_ENDOTHELIAL_GROWTH_FACTOR_PRODUCTION |
| GO_POSITIVE_REGULATION_OF_VASCULAR_ENDOTHELIAL_GROWTH_FACTOR_RECEPTOR_SIGNALING_PATHWAY |
| GO_POSITIVE_REGULATION_OF_VASCULAR_ENDOTHELIAL_GROWTH_FACTOR_SIGNALING_PATHWAY |
| GO_POSITIVE_REGULATION_OF_VASCULAR_SMOOTH_MUSCLE_CELL_DIFFERENTIATION |
| GO_POSITIVE_REGULATION_OF_VASCULAR_SMOOTH_MUSCLE_CELL_PROLIFERATION |
| GO_PURKINJE_MYOCYTE_TO_VENTRICULAR_CARDIAC_MUSCLE_CELL_SIGNALING |
| GO_REGULATION_OF_CARDIAC_EPITHELIAL_TO_MESENCHYMAL_TRANSITION |
| GO_REGULATION_OF_CARDIAC_MUSCLE_ADAPTATION |
| GO_REGULATION_OF_CARDIAC_MUSCLE_CELL_DIFFERENTIATION |
| GO_REGULATION_OF_CARDIAC_MUSCLE_TISSUE_DEVELOPMENT |
| GO_REGULATION_OF_CARDIAC_MUSCLE_TISSUE_REGENERATION |
| GO_REGULATION_OF_CARDIAC_VASCULAR_SMOOTH_MUSCLE_CELL_DIFFERENTIATION |
| GO_REGULATION_OF_CARDIOBLAST_DIFFERENTIATION |
| GO_REGULATION_OF_CARDIOCYTE_DIFFERENTIATON |
| GO_REGULATION_OF_CELL_GROWTH_INVOLVED_IN_CARDIAC_MUSCLE_CELL_DEVELOPMENT |
| GO_REGULATION_OF_CELLULAR_RESPONSE_TO_VASCULAR_ENDOTHELIAL_GROWTH_FACTOR_STIMULUS |
| GO_REGULATION_OF_HEART_RATE_BY_CARDIAC_CONDUCTION |
| GO_STRIATED_MUSCLE_CELL_DIFFERENTIATION |
| GO_STRIATED_MUSCLE_CELL_PROLIFERATION |
| GO_VASCULAR_ASSOCIATED_SMOOTH_MUSCLE_CELL_APOPTOTIC_PROCESS |
| GO_VASCULAR_ASSOCIATED_SMOOTH_MUSCLE_CELL_MIGRATION |
| GO_VASCULAR_ENDOTHELIAL_GROWTH_FACTOR_ACTIVATED_RECEPTOR_ACTIVITY |
| GO_VASCULAR_ENDOTHELIAL_GROWTH_FACTOR_BINDING |
| GO_VASCULAR_ENDOTHELIAL_GROWTH_FACTOR_PRODUCTION |
| GO_VASCULAR_ENDOTHELIAL_GROWTH_FACTOR_RECEPTOR_2_BINDING |
| GO_VASCULAR_ENDOTHELIAL_GROWTH_FACTOR_RECEPTOR_BINDING |
| GO_VASCULAR_ENDOTHELIAL_GROWTH_FACTOR_RECEPTOR_SIGNALING_PATHWAY |
| GO_VASCULAR_ENDOTHELIAL_GROWTH_FACTOR_SIGNALING_PATHWAY |
| GO_VASCULAR_SMOOTH_MUSCLE_CELL_DEVELOPMENT |
| GO_VASCULAR_SMOOTH_MUSCLE_CELL_DIFFERENTIATION |
| GO_VASCULAR_SMOOTH_MUSCLE_CONTRACTION |
| GO_VENTRICULAR_CARDIAC_MUSCLE_CELL_DEVELOPMENT |
| GO_VENTRICULAR_CARDIAC_MUSCLE_CELL_DIFFERENTIATION |
| GO_VENTRICULAR_CARDIAC_MUSCLE_CELL_MEMBRANE_REPOLARIZATION |
| GO_VENTRICULAR_CARDIAC_MUSCLE_TISSUE_DEVELOPMENT |
| GO_VENTRICULAR_COMPACT_MYOCARDIUM_MORPHOGENESIS |
| GO_VENTRICULAR_TRABECULA_MYOCARDIUM_MORPHOGENESIS |
| GO_POSITIVE_REGULATION_OF_BLOOD_CIRCULATION |
| GO_POSITIVE_REGULATION_OF_BLOOD_PRESSURE |
| GO_POSITIVE_REGULATION_OF_BLOOD_PRESSURE_BY_EPINEPHRINE_NOREPINEPHRINE |
| GO_POSITIVE_REGULATION_OF_BLOOD_VESSEL_DIAMETER |
| GO_POSITIVE_REGULATION_OF_BLOOD_VESSEL_ENDOTHELIAL_CELL_MIGRATION |
| GO_POSITIVE_REGULATION_OF_BLOOD_VESSEL_ENDOTHELIAL_CELL_PROLIFERATION_INVOLVED_IN_SPROUTING_ANGIOGENESIS |
| GO_POSITIVE_REGULATION_OF_BLOOD_VESSEL_REMODELING |
| GO_POSITIVE_REGULATION_OF_CELL_MIGRATION_INVOLVED_IN_SPROUTING_ANGIOGENESIS |
| GO_POSITIVE_REGULATION_OF_SYSTEMIC_ARTERIAL_BLOOD_PRESSURE |
| GO_REGULATION_OF_BLOOD_CIRCULATION |
| GO_REGULATION_OF_BLOOD_PRESSURE |
| GO_REGULATION_OF_BLOOD_VESSEL_REMODELING |
| GO_REGULATION_OF_CELL_MIGRATION_INVOLVED_IN_SPROUTING_ANGIOGENESIS |
| GO_REGULATION_OF_SYSTEMIC_ARTERIAL_BLOOD_PRESSURE |
| GO_REGULATION_OF_SYSTEMIC_ARTERIAL_BLOODD_PRESSURE_BY_CIRCULATORY_RENIN_ANGIOTENSIN |
| GO_REGULATION_OF_SYSTEMIC_ARTERIAL_BLOOD_PRESSURE_BY_ENDOTHELIN |
| GO_REGULATION_OF_SYSTEMIC_ARTERIAL_BLOOD_PRESSURE_BY_NOREPINEPHRINE_EPINEPHRINE |
| GO_REGULATION_OF_SYSTEMIC_ARTERIAL_BLOOD_PRESSURE_BY_RENIN_ANGIOTENSIN |
| GO_REGULATION_OF_SYSTEMIC_ARTERIAL_BLOOD_PRESSURE_BY_VASOPRESSIN |
| GO_REGULATION_OF_SYSTEMIC_ARTERIAL_BLOOD_PRESSURE_MEDIATED_BY_A_CHEMICAL_SIGNAL |
| GO_REGULATION_OF_VASOCONSTRICTION |
| GO_RENAL_CONTROL_OF_PERIPHERAL_VASCULAR_RESISTANCE_INVOLVED_IN_REGULATION_OF_SYSTEMIC_ARTERIAL_BLOOD_PRESSURE |
| GO_RENAL_RESPONSE_TO_BLOOD_FLOW_INVOLVED_IN_CIRCULATORY_RENIN_ANGIOTENSIN_REGULATION_OF_SYSTEMIC_ARTERIAL_BLOOD_PRESSURE |
| GO_SPROUTING_ANGIOGENESIS |
| GO_VASCULOGENESIS |
| GO_VENOUS_BLOOD_VESSEL_MORPHOGENESIS |
| GO_VENTRICULAR_CARDIAC_MUSCLE_CELL_DEVELOPMENT |
| GO_NEGATIVE_REGULATION_OF_BLOOD_CIRCULATION |
| GO_NEGATIVE_REGULATION_OF_BLOOD_PRESSURE |
| GO_NEGATIVE_REGULATION_OF_BLOOD_VESSEL_ENDOTHELIAL_CELL_PROLIFERATION_INVOLVED_IN_SPROUTING_ANGIOGENESIS |
| GO_NEGATIVE_REGULATION_OF_BLOOD_VESSEL_ENDOTHELIAL_CELL_MIGRATION |
| GO_NEGATIVE_REGULATION_OF_BLOOD_VESSEL_DIAMETER |
| GO_OUTFLOW_TRACT_MORPHOGENESIS |
| GO_CELLULAR_LIPID_METABOLIC_PROCESS |
| GO_CELLULAR_LIPID_METABOLIC_PROCESS |
| GO_REGULATION_OF_LIPID_METABOLIC_PROCESS |
| **Gene-sets involved in renal development, function, and health** |
| KEGG_ALDOSTERONE_REGULATED_SODIUM_REABSORPTION |
| KEGG_VASOPRESSIN_REGULATED_WATER_REABSORPTION |
| KEGG_ALDOSTERONE_REGULATED_SODIUM_REABSORPTION |
| BIOCARTA_EPONFKB_PATHWAY |
| BIOCARTA_EPONFKB_PATHWAY |
| BIOCARTA_RAS_PATHWAY |
| KEGG_RENIN_ANGIOTENSIN_SYSTEM |
| KEGG_PROXIMAL_TUBULE_BICARBONATE_RECLAMATION |
| REACTOME_REGULATION_OF_WATER_BALANCE_BY_RENAL_AQUAPORINS |
| BIOCARTA_EPO_PATHWAY |
| BIOCARTA_ACE2_PATHWAY |
| PID_EPO_PATHWAY |
| PID_RAS_PATHWAY |
| GO_CELL_DIFFERENTIATION_INVOLVED_IN_KIDNEY_DEVELOPMENT |
| GO_CELL_MIGRATION_INVOLVED_IN_KIDNEY_DEVELOPMENT |
| GO_EPITHELIAL_CELL_DIFFERENTIATION_INVOLVED_IN_KIDNEY_DEVELOPMENT |
| GO_EPITHELIAL_TUBE_MORPHOGENESIS |
| GO_GLOMERULAR_MESANGIAL_CELL_DEVELOPMENT |
| GO_GLOMERULAR_MESANGIAL_CELL_DIFFERENTIATION |
| GO_GLOMERULAR_MESANGIUM_DEVELOPMENT |
| GO_KIDNEY_EPITHELIUM_DEVELOPMENT |
| GO_KIDNEY_MESENCHYME_DEVELOPMENT |
| GO_KIDNEY_MORPHOGENESIS |
| GO_MESANGIAL_CELL_DEVELOPMENT |
| GO_MESENCHYMAL_CELL_DIFFERENTIATION_INVOLVED_IN_KIDNEY_DEVELOPMENT |
| GO_METANEPHRIC_GLOMERULAR_MESANGIUM_DEVELOPMENT |
| GO_METANEPHRIC_RENAL_VESICLE_MORPHOGENESIS |
| GO_METANEPHROS_DEVELOPMENT |
| GO_NEGATIVE_REGULATION_OF_CELL_PROLIFERATION_INVOLVED_IN_KIDNEY_DEVELOPMENT |
| GO_NEGATIVE_REGULATION_OF_EPITHELIAL_CELL_DIFFERENTIATION_INVOLVED_IN_KIDNEY_DEVELOPMENT |
| GO_NEGATIVE_REGULATION_OF_KIDNEY_DEVELOPMENT |
| GO_PATTERN_SPECIFICATION_INVOLVED_IN_KIDNEY_DEVELOPMENT |
| GO_POSITIVE_REGULATION_OF_CELL_PROLIFERATION_INVOLVED_IN_KIDNEY_DEVELOPMENT |
| GO_POSITIVE_REGULATION_OF_EPITHELIAL_CELL_DIFFERENTIATION_INVOLVED_IN_KIDNEY_DEVELOPMENT |
| GO_POSITIVE_REGULATION_OF_KIDNEY_DEVELOPMENT |
| GO_PRONEPHROS_DEVELOPMENT |
| GO_PROXIMAL_DISTAL_PATTERN_FORMATION_INVOLVED_IN_NEPHRON_DEVELOPMENT |
| GO_REGULATION_OF_CELL_PROLIFERATION_INVOLVED_IN_KIDNEY_DEVELOPMENT |
| GO_REGULATION_OF_EPITHELIAL_CELL_DIFFERENTIATION_INVOLVED_IN_KIDNEY_DEVELOPMENT |
| GO_REGULATION_OF_GLOMERULAR_FILTRATION |
| GO_REGULATION_OF_KIDNEY_DEVELOPMENT |
| GO_REGULATION_OF_RENAL_SYSTEM_PROCESS |
| GO_REGULATION_OF_SYSTEMIC_ARTERIAL_BLOOD_PRESSURE_BY_VASOPRESSIN |
| GO_RENAL_ABSORPTION |
| GO_RENAL_CONTROL_OF_PERIPHERAL_VASCULAR_RESISTANCE_INVOLVED_IN_REGULATION_OF_SYSTEMIC_ARTERIAL_BLOOD_PRESSURE |
| GO_RENAL_FILTRATION |
| GO_RENAL_SODIUM_ION_TRANSPORT |
| GO_RENAL_SYSTEM_DEVELOPMENT |
| GO_RENAL_SYSTEM_PROCESS |
| GO_RENAL_SYSTEM_PROCESS_INVOLVED_IN_REGULATION_OF_SYSTEMIC_ARTERIAL_BLOOD_PRESSURE |
| GO_RENAL_SYSTEM_VASCULATURE_DEVELOPMENT |
| GO_RENAL_SYSTEM_VASCULATURE_MORPHOGENESIS |
| GO_RENAL_TUBULE_DEVELOPMENT |
| GO_RENAL_VESICLE_DEVELOPMENT |
| GO_RENAL_VESICLE_FORMATION |
| GO_RENAL_WATER_HOMEOSTASIS |
| GO_RENAL_WATER_TRANSPORT |
| GO_RENAL_SYSTEM_PROCESS_INVOLVED_IN_REGULATION_OF_BLOOD_VOLUME |
| GO_RENAL_SYSTEM_PROCESS_INVOLVED_IN_REGULATION_OF_SYSTEMIC ARTERIAL_BLOOD_PRESSURE |
| GO_RENAL_RESPONSE_TO_BLOOD_FLOW_INVOLVED_IN_CIRCULATORY_RENIN_ANGIOTENSIN_REGULATION_OF_SYSTEMIC_ARTERIAL_BLOOD_PRESSURE |
| GO_RENAL_CONTROL_OF_PERIPHERAL_VASCULAR_RESISTANCE_INVOLVED_IN_REGULATION_OF_SYSTEMIC_ARTERIAL_BLOOD_PRESSURE |
| **Gene-sets involved with the NO-pathway** |
| REACTOME_NITRIC_OXIDE_STIMULATES_GUANYLATE_CYCLASE |
| BIOCARTA_NOS1_PATHWAY |
| BIOCARTA_NO1_PATHWAY |
| REACTOME_CGMP_EFFECTS |
| REACTOME_ENOS_ACTIVATION_AND_REGULATION |
| REACTOME_METABOLISM_OF_NITRIC_OXIDE_ENOS_ACTIVATION_AND_REGULATION |
| GO_NITRIC_OXIDE_MEDIATED_SIGNAL_TRANSDUCTION |
| GO_NITRIC_OXIDE_SYNTHASE_BINDING |
| GO_POSITIVE_REGULATION_OF_NITRIC_OXIDE_SYNTHASE_ACTIVITY |
| GO_CELLULAR_RESPONSE_TO_NITRIC_OXIDE |
| GO_NEGATIVE_REGULATION_OF_NITRIC_OXIDE_METABOLIC_PROCESS |
| GO_NITRIC_OXIDE_MEDIATED_SIGNAL_TRANSDUCTION |
| GO_NITRIC_OXIDE_SYNTHASE_BIOSYNTHETIC_PROCESS |
| GO_NITRIC_OXIDE_SYNTHASE_REGULATOR_ACTIVITY |
| GO_POSITIVE_REGULATION_OF_NITRIC_OXIDE_MEDIATED_SIGNAL_TRANSDUCTION |
| GO_POSITIVE_REGULATION_OF_NITRIC_OXIDE_METABOLIC_PROCESS |
| GO_REGULATION_OF_NITRIC_OXIDE_BIOSYNTHETIC_PROCESS |
| GO_RESPONSE_TO_NITRIC_OXIDE |

These gene-sets were selected from the pre-built gene-sets in homo sapiens in the Molecular Signature database (MsigDB, v7.1), in the hallmark (H) and canonical pathways of curated (C2) collection - containing BioCarta, KEGG, PID, Reactome gene sets – and all gene-sets involved in cardiovascular, renal or NO-pathway of the GO gene-sets (C5) searched for with the terms ‘cardial OR cardiac OR cardio* OR vascular OR blood pressure’, ‘kidney OR renal’, ‘nitric oxide‘^1,2^.

**References**

1. Subramanian A, Tamayo P, Mootha VK, et al. Gene set enrichment analysis: a knowledge-based approach for interpreting genome-wide expression profiles. *Proc Natl Acad Sci U S A*. 2005;102(43):15545-15550.

2. Liberzon A, Birger C, Thorvaldsdóttir H, Ghandi M, Mesirov JP, Tamayo P. The Molecular Signatures Database (MSigDB) hallmark gene set collection. *Cell Syst*. 2015;1(6):417-425.
