# Supplementary material for: Developmental programming in human umbilical cord vein endothelial cells following fetal growth restriction
Source: Clin Epigenetics. 2020 Nov 30;12:185. doi: 10.1186/s13148-020-00980-9 (PMC7708922; doi:10.1186/s13148-020-00980-9)
Supplement: Supplementary file 2 — Additional file 2. Table S2: primers targeting promotor regions. [file 13148_2020_980_MOESM2_ESM.docx]

**Table S2: primers targeting promotor regions**

| Genes | Primers targeting promotor regions |
| --- | --- |
| *FPR3*  (Formyl Peptide Receptor 3) | PCR Forward: 5’- ATTTGGTTAGAAGGATTGTGAAAGTAGAG -3’  PCR Reverse: 5’-Biotin- AAAATCCAAACCCTTTCTTTTCAA -3’  Sequencing: 5’- GGATTGTGAAAGTAGAGAAT -3’  Sequence-to-analyze: YGAGTTTTTG TTTTATYGTG TATTTTTGTT TTTTTATTGT TT |
| *LGALS1*  (Galectin 1) | PCR Forward: 5’- ATGATTGAGTTTAGGAGGATGTT -3’  PCR Reverse: 5’-Biotin- AACCCTATCCTAACTTACAATTAACT -3’  Sequencing: 5’- ATTTTATTAGAGAGAGATGGGTT -3’  Sequence-to-analyze: T TYGGAYGTT TTTATTTTTT TAATTGGATY GGATYGGGTG AGTTTYGTTT TTTGAAAGTT TAGTTAATTG T |
| *NRM*  (Nurim, nuclear envelope membrane protein) | PCR Forward: 5’- GGGGTTATGGAGAGAAATGGAGG -3’  PCR Reverse: 5’-Biotin- CTCTCTCCAAATTCTAACATTCCCTTCTA -3’  Sequencing: 5’- GGTTGAATTTAGTTTAGGAG -3’  Sequence-to-analyze: GGYGGGGTTT TTGTAYGTTA TYGTTAGGTT TTYGGTTYGT TTGGYGTAGT TTTTTTTATT TAGTTGTGGA |
