# Supplementary material for: Developmental programming in human umbilical cord vein endothelial cells following fetal growth restriction
Source: Clin Epigenetics. 2020 Nov 30;12:185. doi: 10.1186/s13148-020-00980-9 (PMC7708922; doi:10.1186/s13148-020-00980-9)
Supplement: Supplementary file 3 — Additional file 3. Figure S1: multidimensional scaling (MDS) plots. [file 13148_2020_980_MOESM3_ESM.docx]

**Figure S1: Multidimensional scaling (MDS) plots**


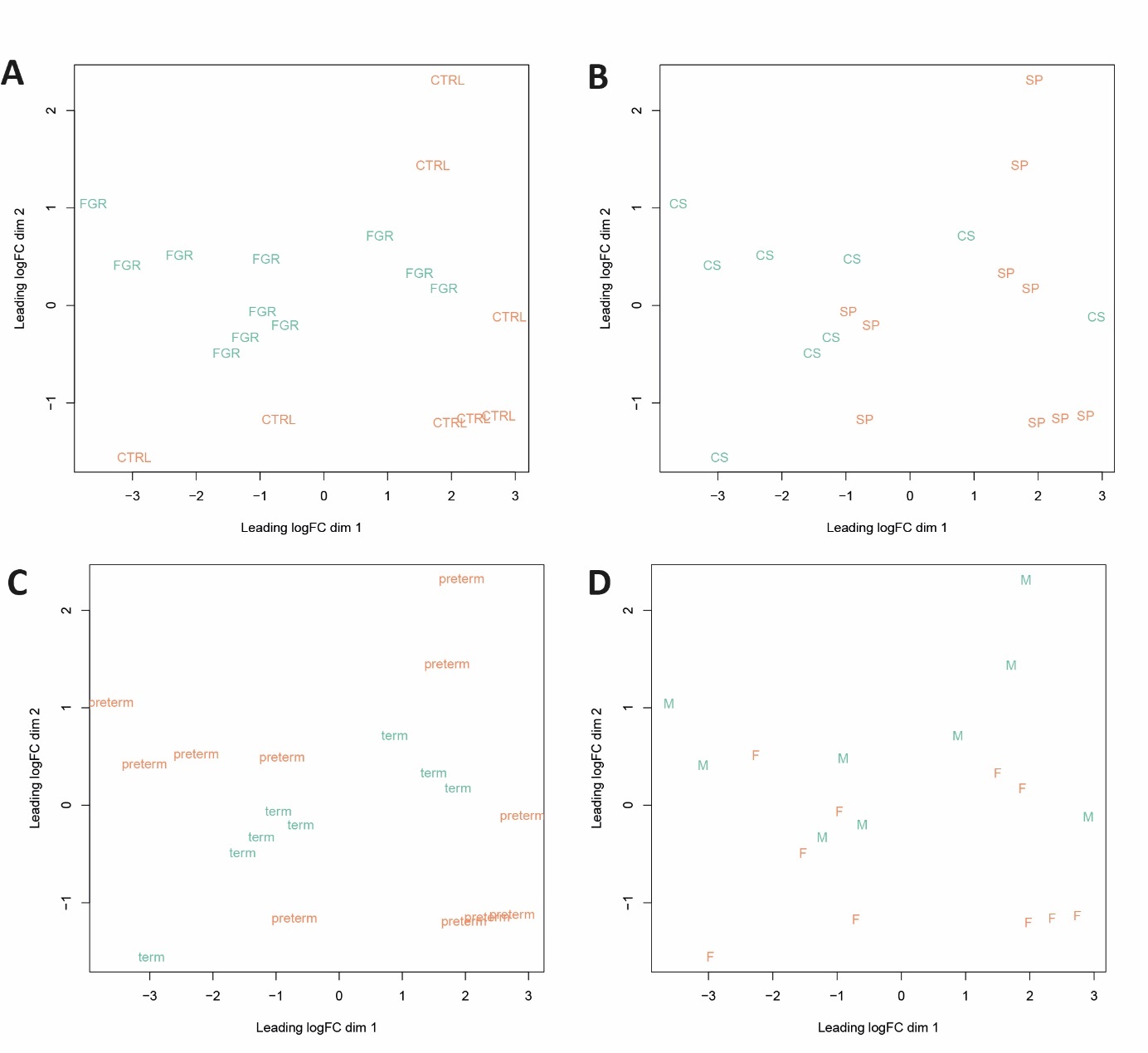


The MDS plots of **A)** study population with fetal growth restriction (FGR) vs control (CTRL), **B)** mode of delivery with caesarian section (CS) vs spontaneous delivery (SP), **C)** gestational age at birth with preterm (<37 weeks) vs term (>37 weeks) and **D)** sex with female (F) vs male (M). Clustering was only visuable for study population and mode of delivery.
