# Supplementary material for: Developmental programming in human umbilical cord vein endothelial cells following fetal growth restriction
Source: Clin Epigenetics. 2020 Nov 30;12:185. doi: 10.1186/s13148-020-00980-9 (PMC7708922; doi:10.1186/s13148-020-00980-9)
Supplement: Supplementary file 4 — Additional file 4. Figure S2: principal component analysis (PCA) plots of fetal growth restriction (FGR) vs. control (CTRL) . [file 13148_2020_980_MOESM4_ESM.docx]

**Figure S2: Principal Component Analysis (PCA) plots of fetal growth restriction (FGR) vs control (CTRL)**


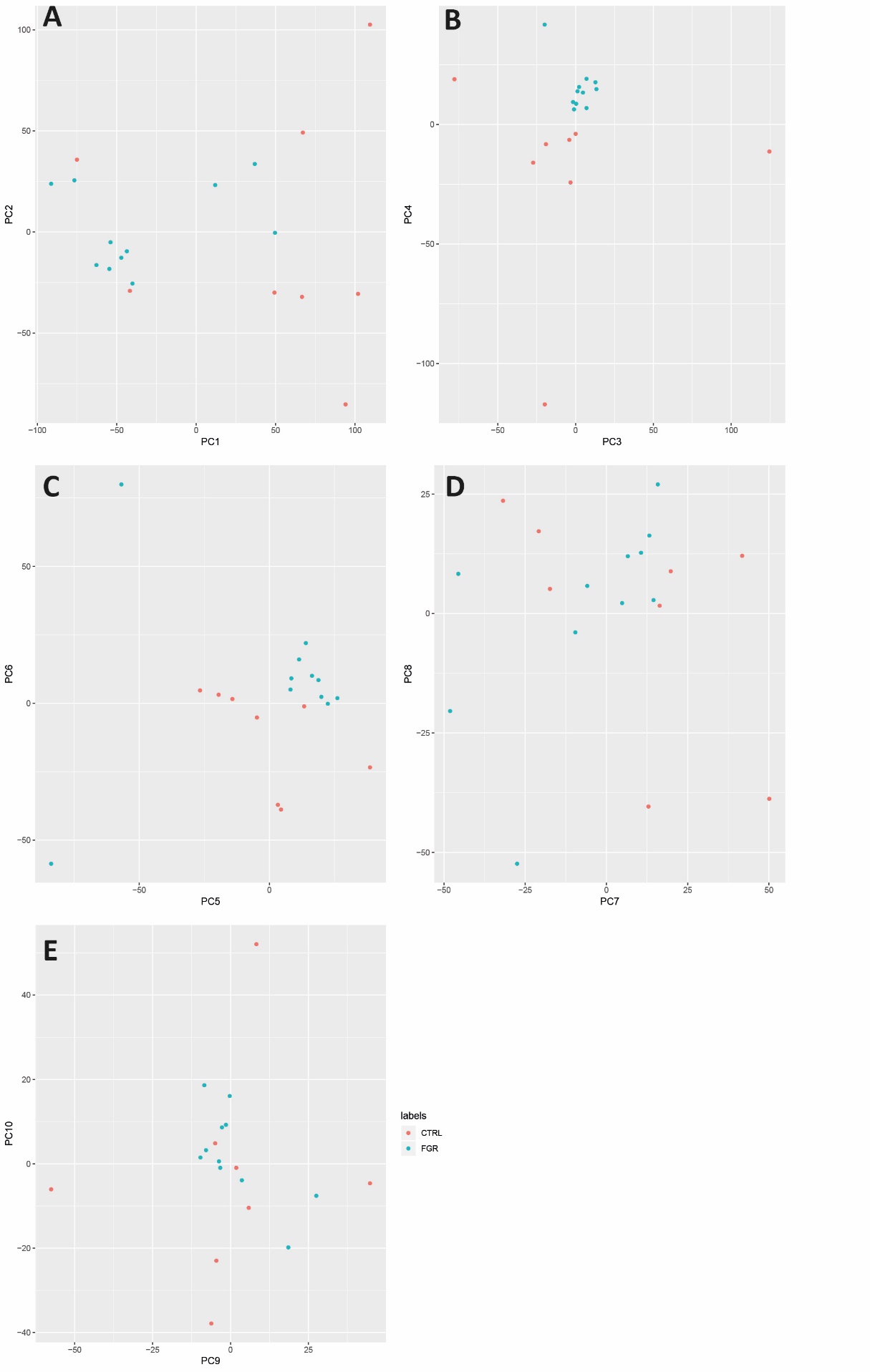


PCA plots of study population of **A)** PC1 vs PC2; **B)** PC3 vs PC4; **C)** PC5 vs PC6; **D)** PC7 vs PC8 and **E)** PC9 vs PC10.
