# Supplementary material for: Developmental programming in human umbilical cord vein endothelial cells following fetal growth restriction
Source: Clin Epigenetics. 2020 Nov 30;12:185. doi: 10.1186/s13148-020-00980-9 (PMC7708922; doi:10.1186/s13148-020-00980-9)
Supplement: Supplementary file 5 — Additional file 5. Table S3: per principal component best correlated modulator. [file 13148_2020_980_MOESM5_ESM.docx]

**Table S3: Per principal component best correlated modulator**

| PC | Modulator | Ranksum | p-value |
| --- | --- | --- | --- |
| PC1 | Delivery route | -2.6128 | **0.009** |
|  | Study Group | -2.4772 | **0.013** |
|  | Gestational Stage | -0.9909 | 0.322 |
|  | Sex | 0.0816 | 1.000 |
| PC2 | Sex | 1.5513 | 0.121 |
|  | Gestational Stage | 0.9093 | 0.364 |
|  | Study Group | 0.9083 | 0.364 |
|  | Delivery route | 0.6532 | 0.514 |
| PC3 | Study Group | 2.1469 | **0.031** |
|  | Gestational Stage | 1.5689 | 0.117 |
|  | Sex | 0.4082 | 0.683 |
|  | Delivery route | 0.1633 | 0.870 |
| PC4 | Study Group | 2.8900 | **0.004** |
|  | Sex | 1.7146 | 0.086 |
|  | Delivery route | 0.8165 | 0.414 |
|  | Gestational Stage | 0.7432 | 0.457 |
| PC5 | Study Group | 1.3211 | 0.186 |
|  | Gestational Stage | 0.8257 | 0.409 |
|  | Sex | 0.4899 | 0.624 |
|  | Delivery route | 0.0000 | 1.000 |
| PC6 | Study Group | 2.3946 | **0.017** |
|  | Gestational Stage | 2.3946 | **0.017** |
|  | Delivery route | 0.4082 | 0.683 |
|  | Sex | -0.1633 | 0.870 |
| PC7 | Delivery route | -2.0412 | **0.041** |
|  | Study Group | -1.3212 | 0.186 |
|  | Gestational Stage | -1.0734 | 0.283 |
|  | Sex | -0.9798 | 0.327 |
| PC8 | Sex | -1.8779 | 0.060 |
|  | Delivery route | -1.1431 | 0.253 |
|  | Gestational Stage | 1.0734 | 0.934 |
|  | Study Group | -0.0826 | 0.934 |
| PC9 | Delivery route | 0.7348 | 0.462 |
|  | Sex | 0.5715 | 0.568 |
|  | Gestational Stage | -0.4954 | 0.620 |
|  | Study Group | -0.1651 | 0.869 |
| PC10 | Gestational Stage | 1.7340 | 0.083 |
|  | Study Group | 1.4863 | 0.137 |
|  | Delivery | 0.5715 | 0.568 |
|  | Sex | 0.0816 | 0.935 |
