# Supplementary material for: Developmental programming in human umbilical cord vein endothelial cells following fetal growth restriction
Source: Clin Epigenetics. 2020 Nov 30;12:185. doi: 10.1186/s13148-020-00980-9 (PMC7708922; doi:10.1186/s13148-020-00980-9)
Supplement: Supplementary file 8 — Additional file 8. Figure S3: heatmaps of significantly differential expressed gene sets involved in cardiovascular or renal development and disease. [file 13148_2020_980_MOESM8_ESM.docx]

**Figure S3: Heatmaps of significantly differential expressed gene sets involved in cardiovascular or renal development and disease**


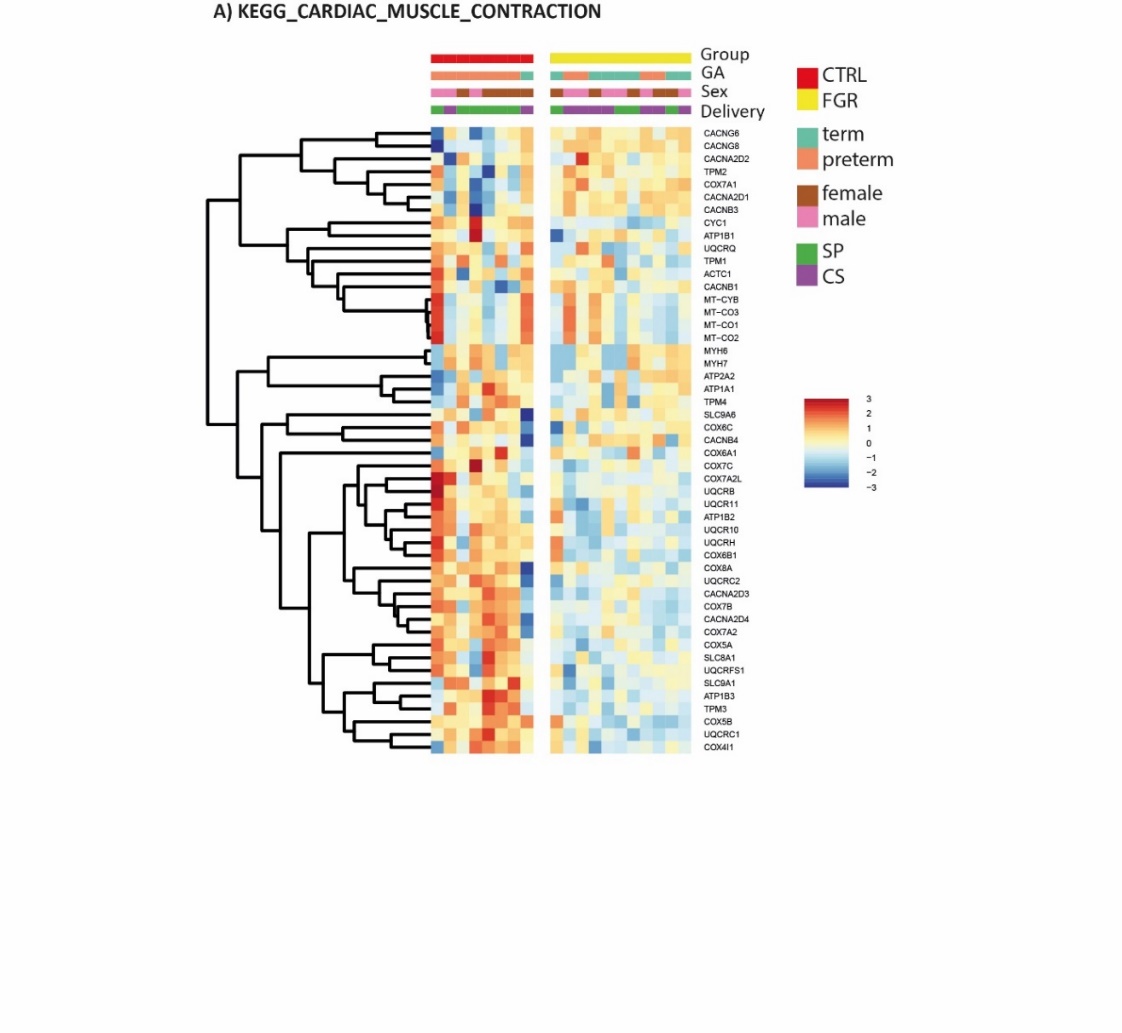


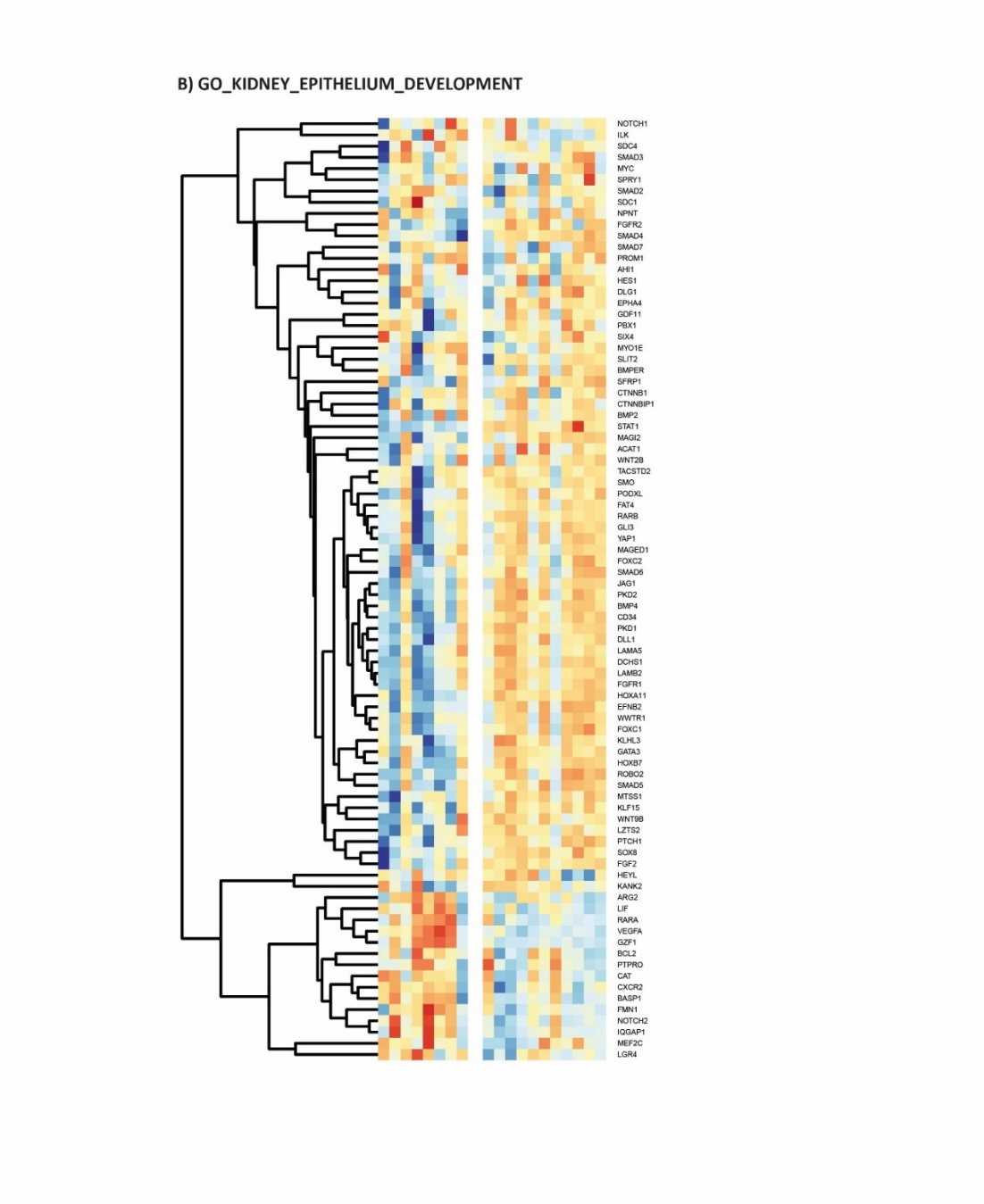


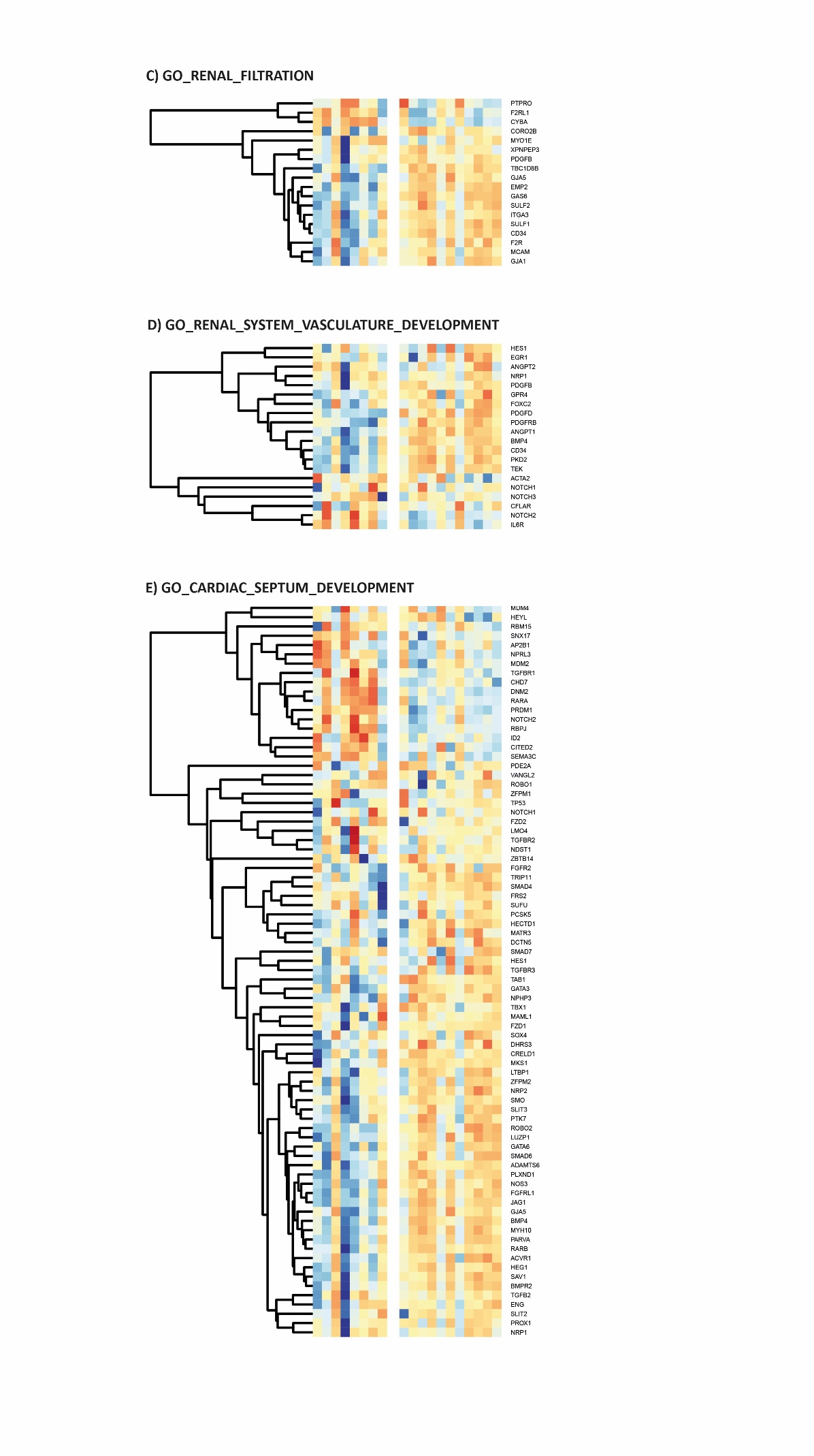


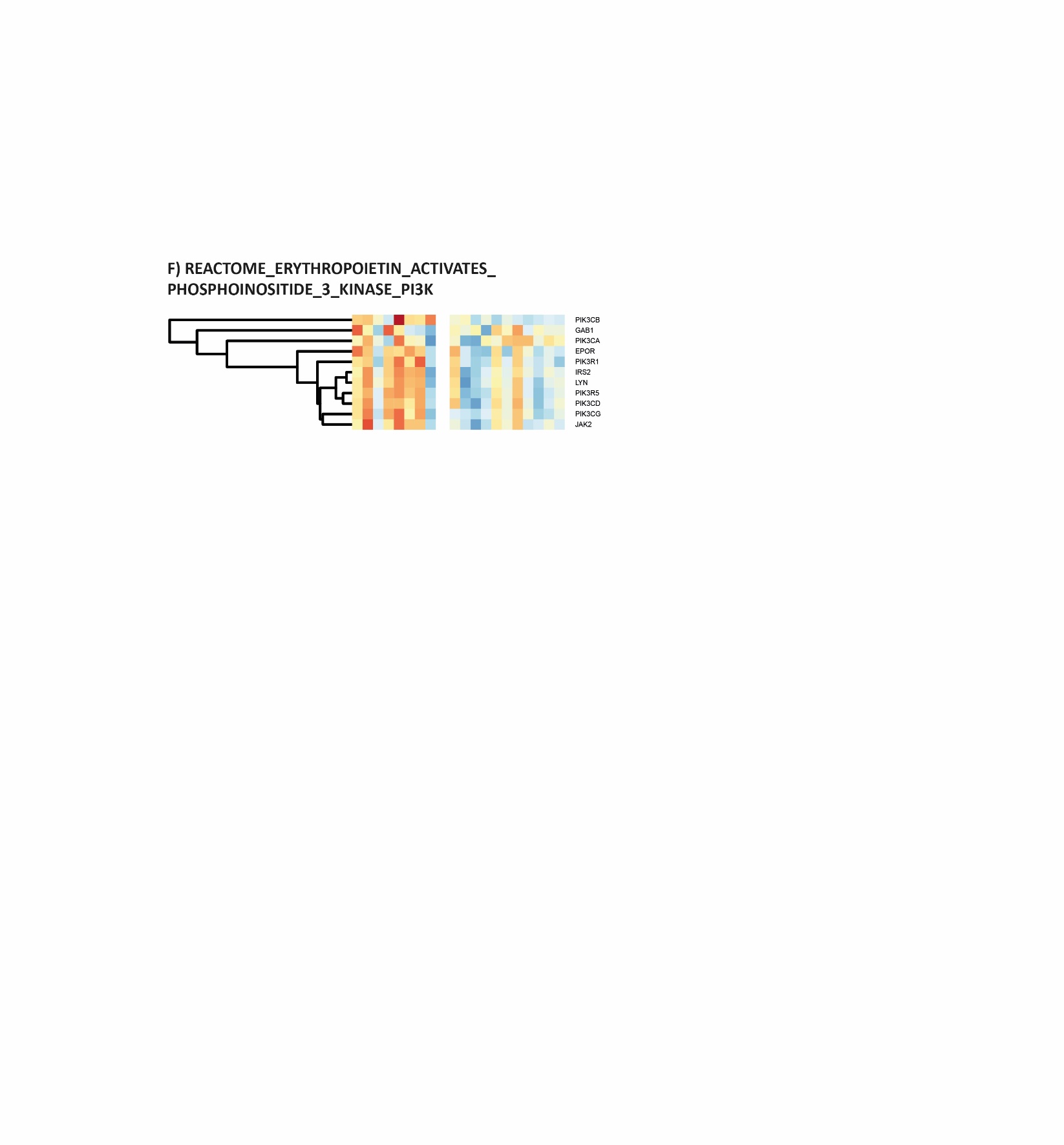


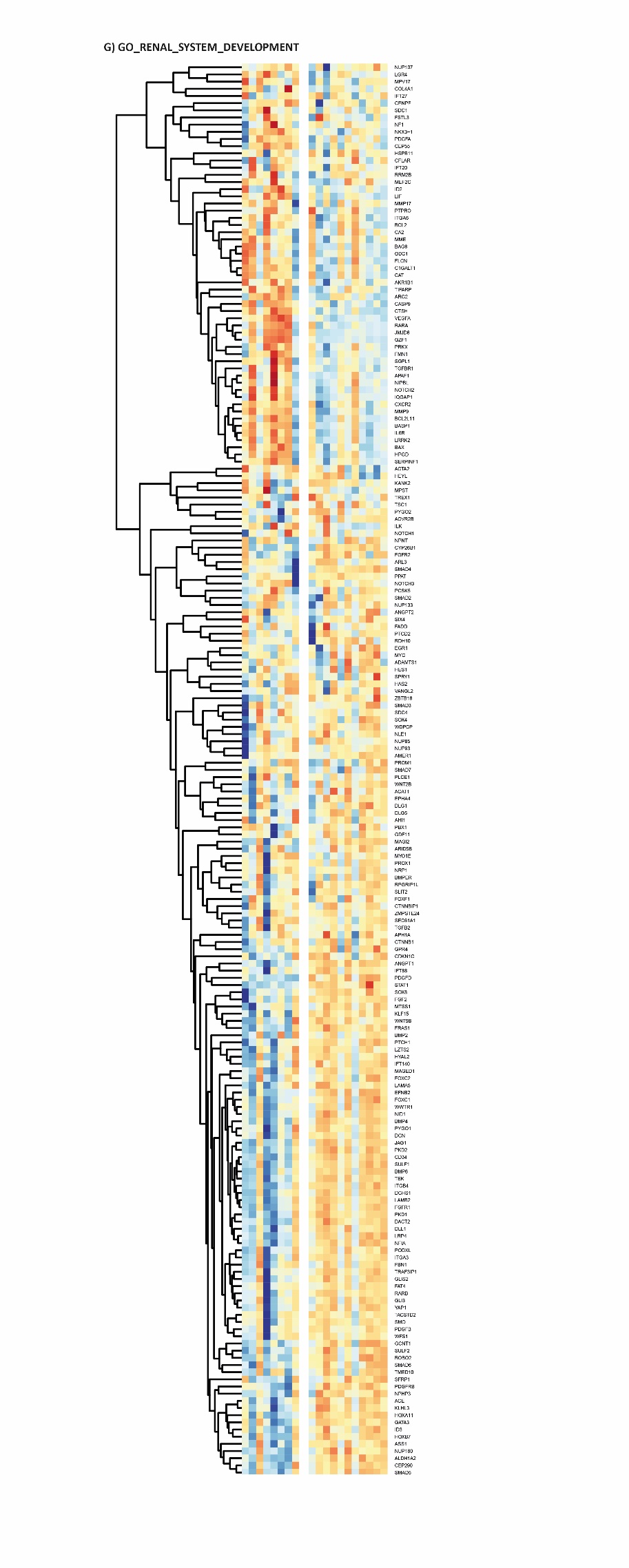


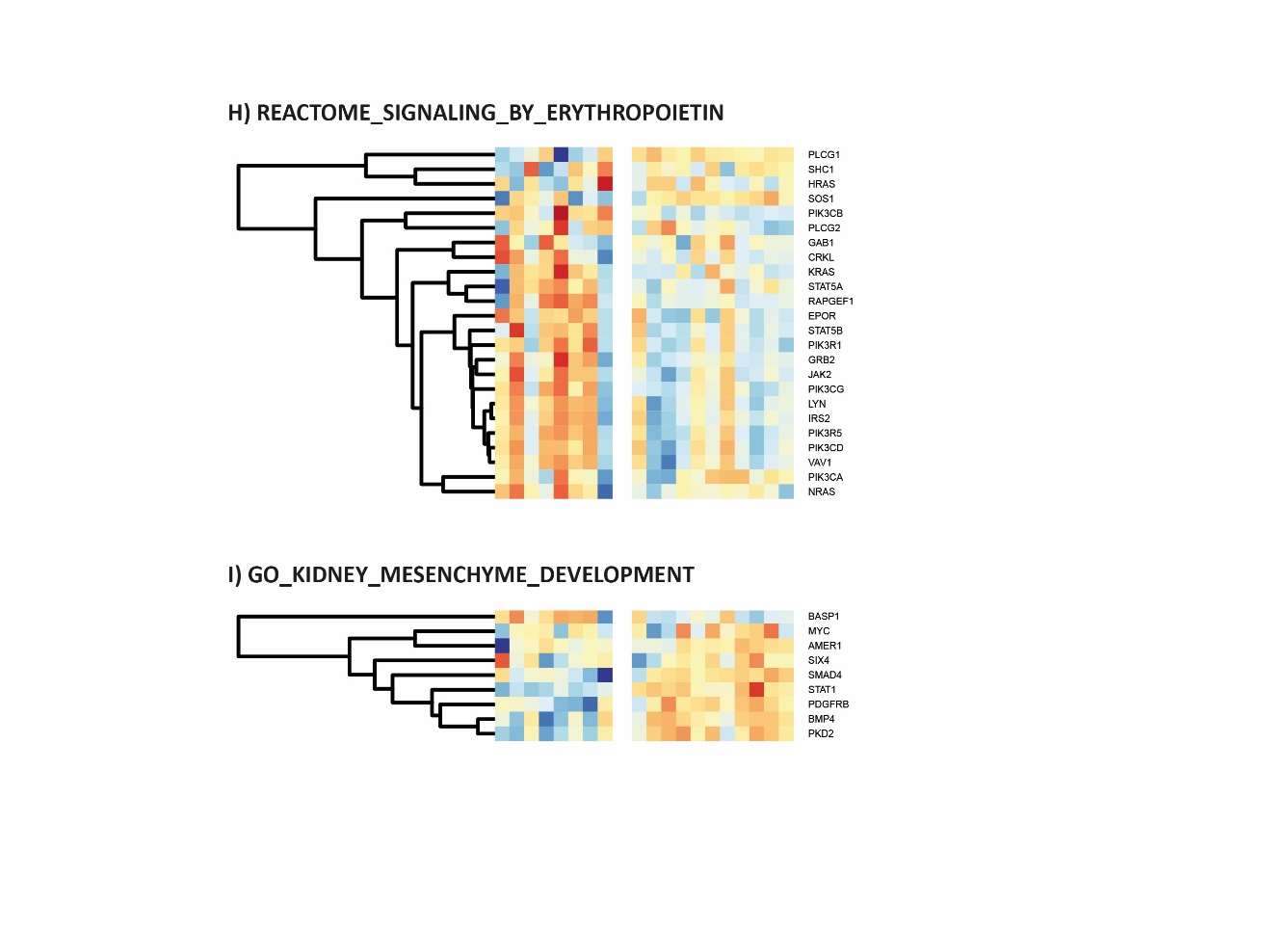


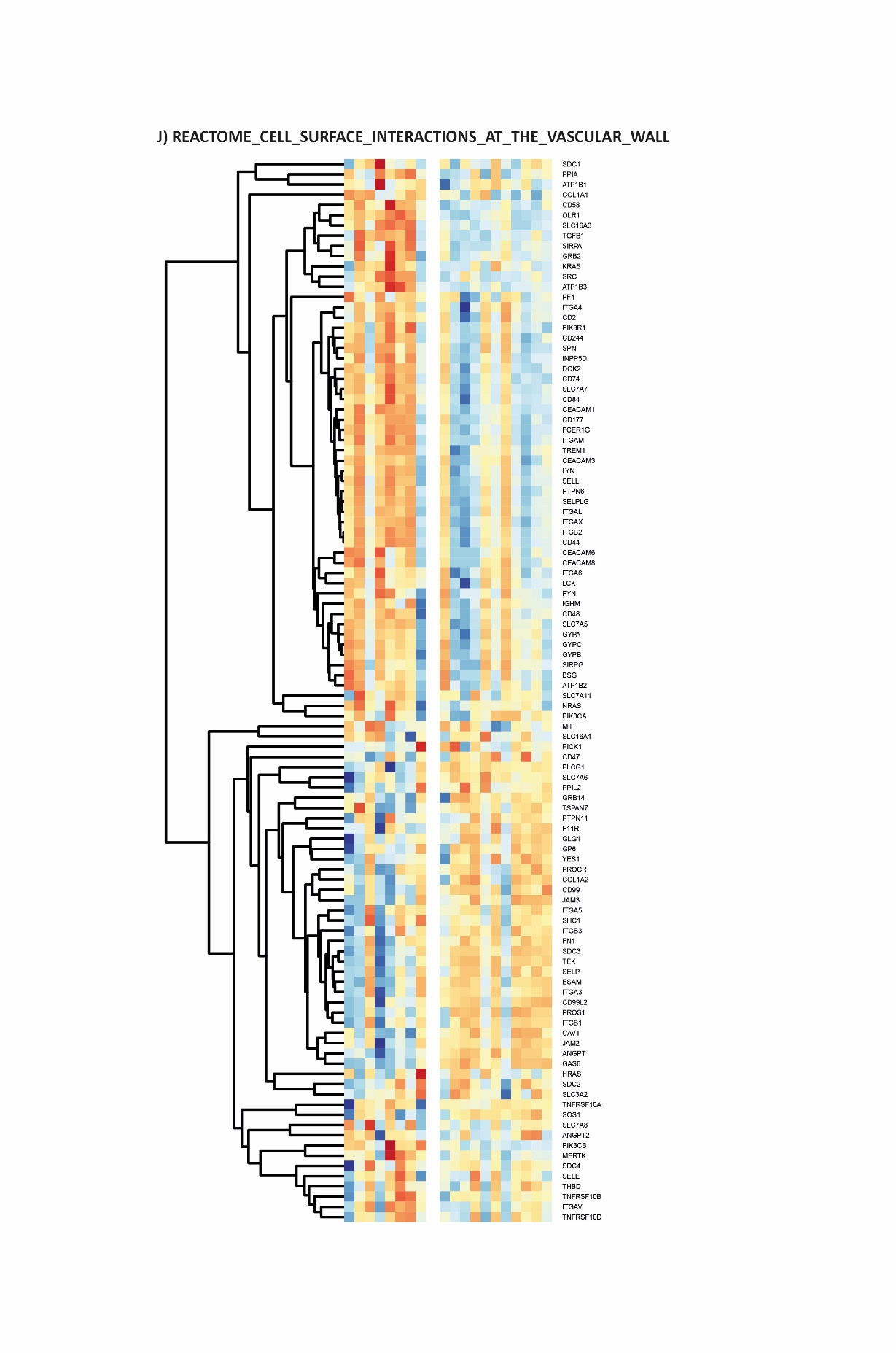

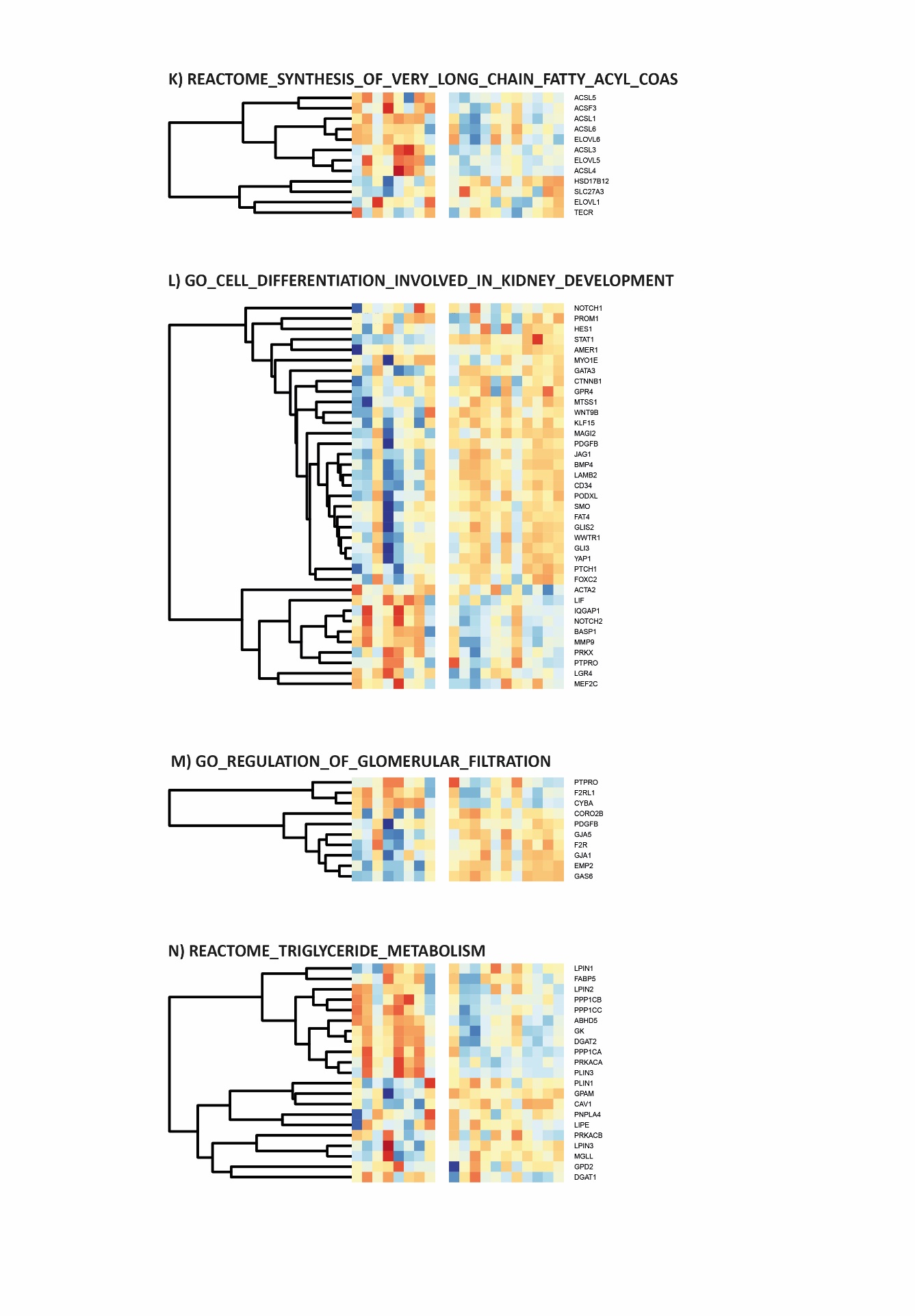


CS, caesarean section; CTRL, control; FGR, fetal growth restriction; SP, spontaneous delivery.
