# Supplementary material for: Developmental programming in human umbilical cord vein endothelial cells following fetal growth restriction
Source: Clin Epigenetics. 2020 Nov 30;12:185. doi: 10.1186/s13148-020-00980-9 (PMC7708922; doi:10.1186/s13148-020-00980-9)
Supplement: Supplementary file 9 — Additional file 9. Figure S4: DNA methylation at individual CpG positions for FPR3. [file 13148_2020_980_MOESM9_ESM.docx]

**Figure S4: DNA methylation at individual CpG positions for *FPR3***

**
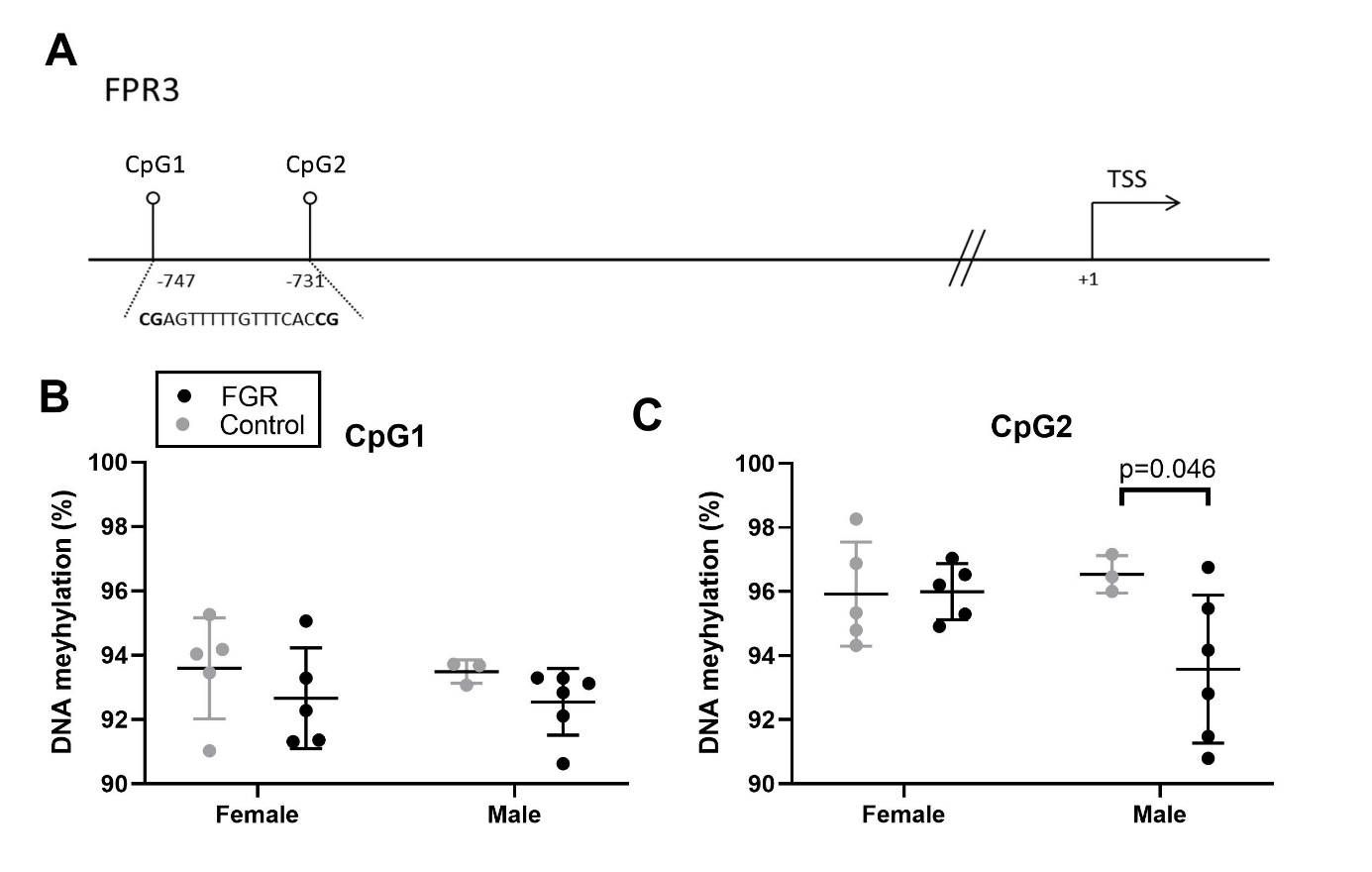
A)** The examined CpG positions in relation to the transcription start site (TSS); **B)** DNA methylation at CpG1; **C)** DNA methylation at CpG2 in fetal growth restriction (FGR) (n=11) vs control (n=8). Data shown as Mean ± SD. Tested with 2-way ANOVA with Bonferroni multiple comparison. *FPR3,* formyl peptide receptor 3.
